# CCR1-mediated monocyte chemotaxis in the immunopathology of primary Sjögren’s syndrome: multi-omics integration analysis and computational target prioritization implicating *Polygonatum odoratum*

**DOI:** 10.3389/fimmu.2026.1867098

**Published:** 2026-07-20

**Authors:** Jianbin Li, Yuzhen Gesang, Ning Tan, Renhe Li, Wei Liu

**Affiliations:** 1 Department of Rheumatology and Immunology, First Teaching Hospital of Tianjin University of Traditional Chinese Medicine, Tianjin, China; 2 National Clinical Research Center for Chinese Medicine, Tianjin, China; 3 Department of Rheumatism and Immunity, Shenzhen Nanshan People’s Hospital, Shenzhen, China

**Keywords:** CCR1, chemokines, monocytes, *Polygonatum odoratum*, primary Sjögren’s syndrome, pseudotime analysis, SCENIC, single-cell transcriptomics

## Abstract

**Objective:**

Primary Sjögren’s syndrome (pSS) is a chronic systemic autoimmune disease with no specific etiological treatment. *Polygonatum odoratum*, a traditional yin-nourishing herb, is used in treating pSS, but its molecular mechanism remains unclear. This study aimed to explore the potential association of CCR1 with pSS immunopathology and to computationally assess its candidacy as a predicted target of P. odoratum.

**Methods:**

Transcriptomic datasets (GSE51092, GSE66795; validation: GSE84844) and single-cell RNA sequencing data (GSE253568) were obtained from GEO. Network pharmacology screened active component targets of P. odoratum, and a “three-dimensional intersection” strategy identified candidate therapeutic targets. Single-cell analyses including monocyte subtyping, pseudotime trajectory, SCENIC transcription factor network, and CellChat cell communication analyses were performed. Molecular docking predicted drug-target binding, and RT-qPCR validated findings in clinical samples (pSS n = 65, HC n = 48).

**Results:**

CCR1 was the only candidate target simultaneously satisfying “drug-targetable, disease-related, and differentially expressed” conditions. CCR1 was significantly upregulated in pSS patients (AUC = 0.758, indicating moderate discriminatory capacity) and positively correlated with serum IgG levels (R = 0.49, P = 0.0057). Single-cell analysis demonstrated CCR1 was specifically highly expressed in classical monocytes (CD14++CD16−), with CCR1-positive monocytes significantly expanded in pSS (P = 0.0079). SCENIC analysis predicted STAT1 and EGR1 as candidate upstream transcription factors potentially associated with CCR1 expression. CellChat analysis predicted a potential “T/NK cell-derived CCL5–CCR1-mediated monocyte chemotactic recruitment” signaling axis. Moupinamide from P. odoratum showed predicted binding affinity with CCR1 (−8.1 kcal/mol). RT-qPCR confirmed elevated CCR1 expression in pSS PBMCs (P < 0.0001).

**Conclusions:**

This study provides exploratory multi-dimensional evidence consistent with a candidate role of CCR1 in pSS immunopathology. CCR1 is predominantly expressed in classical monocytes, predicted to be associated with STAT1/EGR1 regulatory activity, and computationally linked to monocyte recruitment through the CCL5–CCR1 chemotactic axis. Pseudotime analysis suggested a computational inference of monocyte differentiation trajectory differences associated with CCR1 expression dynamics, requiring experimental validation. Moupinamide from P. odoratum shows predicted binding affinity to CCR1, offering a computational hypothesis for the “yin-nourishing and dryness-moistening” therapeutic principle that warrants experimental validation.

## 1 Introduction

Primary Sjögren’s syndrome (pSS) is a chronic systemic autoimmune disease characterized by lymphocytic infiltration and progressive destruction of exocrine glands, primarily the salivary and lacrimal glands (1). Global epidemiological surveys indicate that the prevalence of pSS is approximately 0.1%–0.5%, with significantly higher incidence in females, and peak onset between ages 40–60 (2). The clinical manifestations of pSS are highly heterogeneous; beyond typical xerostomia and xerophthalmia, approximately 60%–80% of patients develop extraglandular organ involvement, severely impacting quality of life and prognosis (3). Notably, pSS patients have a 15–20 fold increased risk of developing non-Hodgkin lymphoma (4).

The pathogenesis of pSS involves complex interactions among genetic susceptibility, environmental triggers, and immune dysregulation (5). At the immunological level, activation of the type I interferon signature is considered a core event in pSS pathogenesis, inducing upregulation of interferon-stimulated genes and subsequently driving B cell hyperactivation, autoantibody production, and glandular inflammatory damage (6). The monocyte/macrophage system plays an important role in innate immune responses and inflammation maintenance in pSS, but the specific molecular regulatory mechanisms remain incompletely understood (7). Currently, pSS treatment relies primarily on symptomatic management and nonspecific immunosuppressants, lacking targeted therapies addressing the underlying etiology (8).

Chemokines and their receptors play central regulatory roles in the development of autoimmune diseases (9). C-C chemokine receptor type 1 (CCR1) is an important member of the CC chemokine receptor family, primarily expressed on monocytes, macrophages, and other immune cells, participating in chemotactic recruitment of inflammatory cells to sites of injury (10). Previous studies have shown that CCR1 is upregulated in autoimmune diseases such as rheumatoid arthritis and multiple sclerosis, and CCR1 antagonists have demonstrated good anti-inflammatory effects in animal models (11). However, systematic studies on CCR1 expression characteristics in pSS and its feasibility as a drug target remain lacking.

*Polygonatum odoratum* (Mill.) Druce, the dried rhizome of a perennial herbaceous plant in the Polygonatum genus of the Liliaceae family, is one of China’s traditional medicine-food homologous species. According to traditional Chinese medicine theory, pSS belongs to the category of “dryness syndrome,” and treatment should focus on nourishing yin, promoting fluid production, and moistening dryness. P. odoratum has a slightly cold nature and sweet taste, enters the lung and stomach meridians, and possesses the effects of nourishing yin and moistening dryness. Modern pharmacological studies have shown that P. odoratum contains abundant bioactive components including steroidal saponins, flavonoids, alkaloids, and polysaccharides, with anti-inflammatory and immunomodulatory activities (12). Exploring the molecular mechanisms of P. odoratum in treating pSS holds significant scientific and clinical value.

This study employed a multi-omics integration strategy, computationally prioritizing CCR1 as a candidate target of P. odoratum for treating pSS from four dimensions: transcriptomics, network pharmacology, single-cell sequencing, and clinical sample validation. Furthermore, monocyte subtype characterization, pseudotime trajectory analysis, SCENIC transcription factor network, and CCL pathway cell communication analysis were conducted to elucidate the role of CCR1 in pSS monocyte immunopathology, generating testable hypotheses for potential therapeutic strategies in pSS.

## 2 Materials and methods

### 2.1 Transcriptomic data sources and preprocessing

The pSS transcriptomic data used in this study were publicly obtained from the NCBI Gene Expression Omnibus (GEO) database. Training datasets included two independent cohorts: GSE51092 (190 cases and 32 controls in total; from which we selected 30 pSS patients meeting 2002 AECG criteria and 17 age- and sex-matched healthy controls based on complete clinical annotation and diagnostic confirmation; peripheral whole blood; Illumina HumanWG-6 v3.0 expression beadchip) and GSE66795 (131 pSS patients and 29 healthy controls; peripheral whole blood; Illumina HumanHT-12 V4.0). GSE84844 (30 pSS patients and 30 healthy controls; GPL570 Affymetrix HG-U133 Plus 2.0) served as an independent external validation dataset. Single-cell RNA sequencing data GSE253568 contained PBMC samples from 9 pSS patients and 8 healthy controls.Data preprocessing: raw expression matrices underwent log2 transformation; quantile normalization was applied; the ComBat function in the R package sva (version 3.44.0) was used for batch effect correction (13, 14). Batch correction effectiveness was evaluated through PCA visualization. The complete list of GEO sample identifiers (GSM accessions) used in each dataset, along with group assignments and sample-selection criteria, is provided in **Supplementary Table 10**. Platform-specific probe-to-gene annotation was performed using the illuminaHumanv3.db package for GSE51092, the illuminaHumanv4.db package for GSE66795, and the hgu133plus2.db package for GSE84844. For probes mapping to multiple genes, the probe with the highest mean expression was retained. The full analysis code and processed expression matrices are provided as the **Supplementary Code File** (see Data Availability Statement). Following correction of the dataset metadata to match the GEO public records, we re-ran the complete analysis pipeline and confirmed that all principal findings were reproduced without substantive changes.

### 2.2 Collection of Sjögren’s syndrome disease targets

pSS-related disease genes were comprehensively collected from four databases: (1) GeneCards (version 5.12): genes with Relevance score > 10; (2) OMIM: all gene entries related to Sjögren syndrome; (3) DisGeNET (version 7.0): genes with Gene-Disease Association Score > 0.1; (4) TTD (updated to 2022): drug target genes related to Sjögren’s syndrome. Gene symbols were standardized using the UniProt database.

### 2.3 Screening of active components and target prediction of P. odoratum

Chemical component information of P. odoratum was obtained from the HERB 2.0 database. SwissADME was used to evaluate pharmacokinetic properties of each candidate compound. Screening criteria: (1) Gastrointestinal absorption assessed as “High”; (2) Compliance with Lipinski’s Rule of Five with no more than one violation (molecular weight ≤ 500 Da, calculated LogP ≤ 5, hydrogen bond donors ≤ 5, hydrogen bond acceptors ≤ 10); compounds violating two or more of Lipinski’s rules were excluded to ensure adequate predicted oral bioavailability; (3) Simultaneous compliance with Veber’s rules (topological polar surface area [TPSA] ≤ 140 Å², number of rotatable bonds ≤ 10). A total of 24 compounds passed all three screening criteria and were retained for subsequent target prediction (**Supplementary Table 9**). SwissTargetPrediction was used for target prediction (species: Homo sapiens, Probability > 0).

### 2.4 Differential expression gene analysis

The R package limma (version 3.52.4) was used for differential expression analysis (15). DEG screening criteria: |log2FoldChange| > 0.585 and adjusted P value < 0.05, with Benjamini-Hochberg correction for multiple testing.

### 2.5 Functional enrichment analysis

The R package clusterProfiler (version 4.4.4) was used for GO and KEGG pathway enrichment analysis (16), with pAdjustMethod = “BH” and pvalueCutoff = 0.05.

### 2.6 Candidate therapeutic target screening strategy

A “three-dimensional intersection” strategy was adopted: intersection of P. odoratum active component targets, pSS disease genes, and DEGs. Genes simultaneously meeting all three conditions were identified as candidate targets for further investigation.

### 2.7 Diagnostic model construction and evaluation

A nomogram diagnostic model was constructed using the R package rms. Performance evaluation included ROC curves (AUC), calibration curves (MAE), decision curve analysis (DCA), and clinical impact curves (CIC).

### 2.8 Gene set enrichment analysis

Samples were divided into high and low expression groups based on median expression of the candidate target. The GSEA function in clusterProfiler was used with the MSigDB reference gene set c2.cp.kegg.v7.4.symbols.gmt (17). Parameters: nPerm = 1000, minGSSize = 10, maxGSSize = 500, pvalueCutoff = 0.05.

### 2.9 Immune cell infiltration analysis

The CIBERSORT algorithm was used with the LM22 signature matrix for deconvolution analysis (18). Only samples with P value < 0.05 (1000 permutations) were retained. GSVA (version 1.44.2) was used for pathway activity analysis.

### 2.10 Single-cell RNA sequencing data analysis

Single-cell data (GSE253568) were processed using the Seurat R package (version 5.0) (19). Quality control: genes per cell 200–5000, mitochondrial gene percentage < 15%, UMI counts > 500. NormalizeData (LogNormalize, scale.factor = 10000), FindVariableFeatures (vst, nfeatures = 1500), ScaleData, RunPCA (npcs = 20), Harmony batch correction, RunTSNE visualization, FindClusters (resolution = 0.6). Cell type annotation was performed using SingleR with multiple reference datasets.

### 2.11 Monocyte subtype characterization

After extracting the monocyte subpopulation, re-normalization, variable feature identification, and sub-clustering were performed. RunPCA (npcs = 20), RunHarmony, RunUMAP (dims = 1:15), FindClusters (resolution = 0.4). Based on CD14 and FCGR3A (CD16) expression patterns, combined with subtype-specific markers (S100A8/A9/A12 for classical; HLA-DRA/CSF1R/CD163 for intermediate; MS4A7/CDKN1C for non-classical), monocytes were classified into three functional subtypes: classical (CD14++CD16−), intermediate (CD14++CD16+), and non-classical (CD14+CD16++). FindAllMarkers was used to identify cluster-specific marker genes.

### 2.12 Milo differential abundance analysis

The miloR R package was used for differential abundance analysis (20). Milo constructs cell neighborhoods based on KNN graphs (k = 30) and tests for differential abundance between conditions (design = ~Type) without requiring pre-defined cluster assignments. SpatialFDR correction was applied to assess enrichment direction of each neighborhood in pSS.

### 2.13 Slingshot pseudotime analysis

The slingshot R package was used for trajectory inference (21). Seurat objects were converted to SingleCellExperiment objects. The cluster enriched for classical monocytes served as the starting point. Slingshot reconstructed continuous differentiation trajectories using minimum spanning trees and simultaneous principal curves. GAM models were used to fit expression dynamics of CCR1 and other key genes along the pseudotime axis.

### 2.14 SCENIC transcription factor regulatory network analysis

The SCENIC R package was used (22). Workflow: (1) GENIE3 for gene co-expression module inference; (2) RcisTarget with hg38 mc9nr feather databases for motif enrichment and regulon identification; (3) AUCell for regulon activity scoring per cell. Wilcoxon tests compared regulon activity between pSS and control, and Spearman correlations were calculated between regulon activity and CCR1 expression.

### 2.15 Cell-cell communication analysis

The CellChat R package (version 1.6.1) was used (23). Separate CellChat objects were constructed for pSS and control groups using the “Secreted Signaling” subset of CellChatDB.human. computeCommunProb calculated communication probability, and filterCommunication filtered low-confidence signals (min.cells = 10). netVisual_contribution evaluated relative contributions of ligand-receptor pairs in the CCL pathway.

### 2.16 Virtual gene knockout analysis

The scTenifoldKnk algorithm was used to simulate CCR1 knockout effects in monocytes from single-cell datasets (24). This method constructs wild-type and virtual knockout gene regulatory networks, using tensor decomposition and manifold alignment to calculate expression change scores for each gene.

### 2.17 Competing endogenous RNA network construction

A lncRNA-miRNA-mRNA ceRNA network centered on CCR1 was constructed. miRNA prediction: intersection of miRDB, miRTarBase, and TargetScan results. lncRNA prediction: ENCORI/starBase with CLIP Data ≥ 1 and Degradome Data ≥ 1. Visualization: Cytoscape (version 3.9.1).

### 2.18 Molecular docking

AutoDock Vina (version 1.2.0) was used for molecular docking (25) with exhaustiveness = 32. Binding energy < −5.0 kcal/mol indicated good binding activity, < −7.0 kcal/mol indicated strong binding activity. Results were visualized using PyMOL and Discovery Studio Visualizer.

### 2.19 Clinical sample collection and RT-qPCR validation

This study was approved by the Ethics Committee of the First Teaching Hospital of Tianjin University of Traditional Chinese Medicine (TYLL2018[K]026). All participants provided written informed consent. pSS patients met the 2016 ACR/EULAR classification criteria (26). PBMCs were isolated using Ficoll density gradient centrifugation. Total RNA was extracted using TRIzol (Invitrogen). RT-qPCR was performed using TB Green Premix Ex Taq II (TaKaRa). Primers: CCR1 forward 5’-CAACTCCGTGCCAGAAGGTGAA-3’, reverse 5’-GTTCAGGAGGTAGATGCTGGTC-3’; GAPDH forward 5’-GAAGGTGAAGGTCGGAGTC-3’, reverse 5’-GAAGATGGTGATGGGATTTC-3’. Relative expression was calculated using the 2^(−ΔΔCt) method.

### 2.20 Statistical analysis

All statistical analyses were performed using R (version 4.2.1) and GraphPad Prism (version 9.0). Continuous variables were compared using independent t-tests (normally distributed) or Wilcoxon rank-sum tests (non-normally distributed). Correlation analysis used Spearman’s rank correlation. Diagnostic performance was evaluated using ROC curves. All tests were two-sided, with P < 0.05 considered statistically significant.

## 3 Results

### 3.1 Identification and integration analysis of key differentially expressed genes in Sjögren’s syndrome

**Figure 1:**
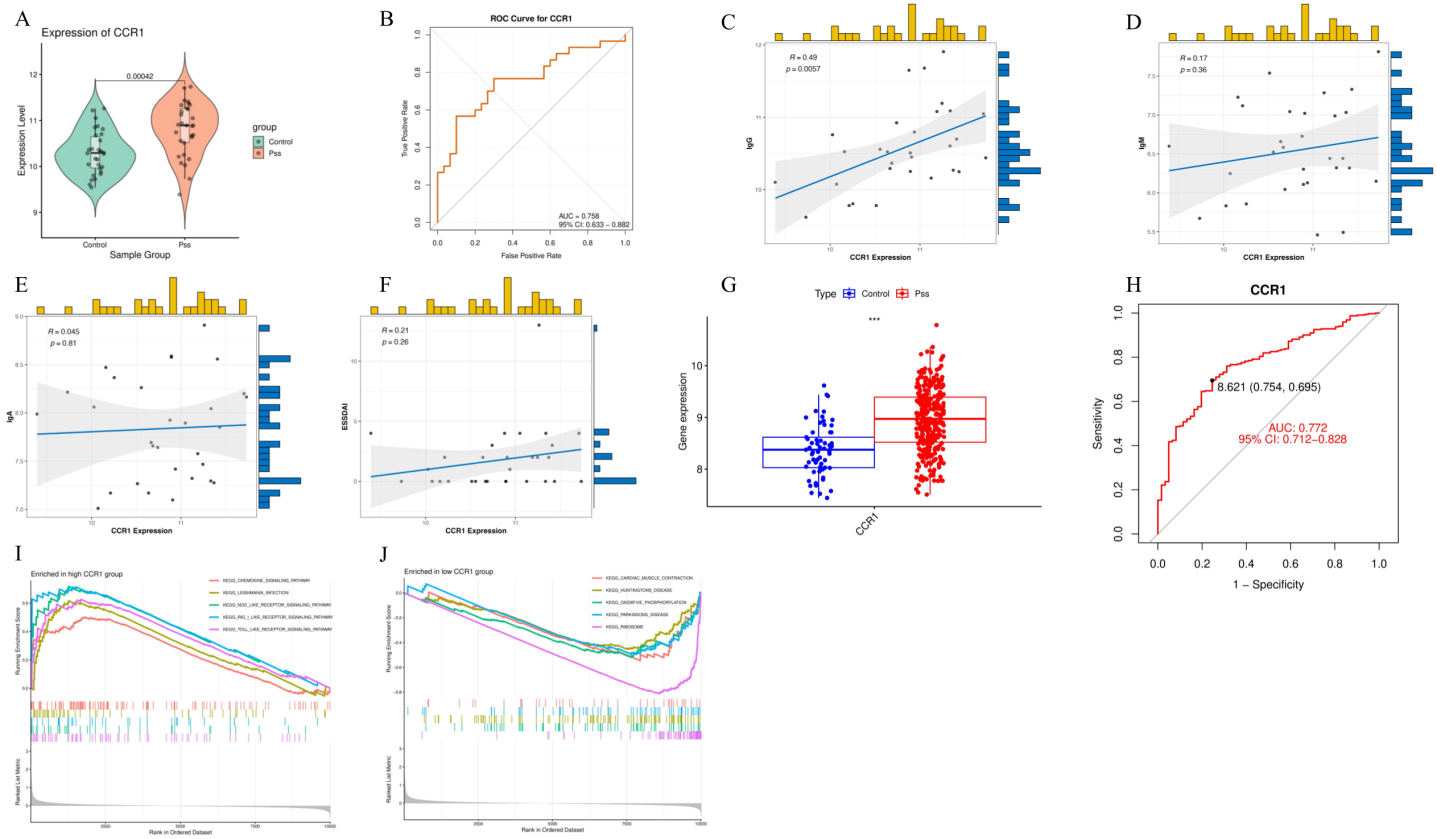
Identification of differentially expressed genes in pSS, network pharmacology analysis, and identification of candidate target CCR1. **(A)** PCA plot after batch effect removal, showing good mixing of samples from two datasets; **(B)** Volcano plot of differentially expressed genes, red indicates upregulated genes (e.g., IFI44L), blue indicates downregulated genes (e.g., PTGDS), gray indicates no difference; **(C)** Heatmap of significantly differentially expressed genes, red represents high expression, blue represents low expression; **(D)** Venn diagram of (P) odoratum active component targets and pSS disease targets, identifying 69 intersection targets; **(E)** PPI network of intersection targets; **(F)** GO functional enrichment analysis bar chart showing enriched terms for Biological Process (BP), Cellular Component (CC), and Molecular Function (MF); **(G)** KEGG pathway enrichment analysis bubble chart, bubble size represents number of enriched genes, color represents P value significance; **(H)** Venn diagram of pSS-related disease targets retrieved from GeneCards, OMIM, DisGeNET, and TTD databases; **(I)** PPI network of potential key targets, node size and color depth reflect degree value; **(J)** Three-dimensional intersection Venn diagram of “drug targets-disease targets-DEGs,” arrow points to the only overlapping gene—CCR1.

To overcome single-center data bias, this study incorporated and integrated the GSE51092 and GSE66795 datasets. After batch effect removal using the ComBat algorithm, principal component analysis (PCA) showed uniform mixing of samples between groups without obvious technical bias (**Figure 1A**). Based on criteria of |log2FC| > 0.585 and adj. P < 0.05, we identified a total of 85 differentially expressed genes (DEGs), including 69 upregulated and 16 downregulated genes. The volcano plot (**Figure 1B**) showed that interferon-related genes such as IFI44L and RSAD2 were significantly upregulated in pSS patients, while genes including PTGDS and FEZ1 were significantly downregulated. The heatmap of differentially expressed genes (**Figure 1C**) further verified that these genes could clearly distinguish pSS patients from healthy controls. Notably, chemokine receptor CCR1 showed significant high expression in the pSS group, suggesting its potential involvement in disease-associated immune processes.

### 3.2 Identification and functional enrichment analysis of potential targets of P. odoratum for treating pSS

We mapped the screened P. odoratum active component targets with pSS-related disease targets. Venn diagram results showed (**Figure 1D**) 69 common targets between them, considered potential key effector molecules of P. odoratum for treating pSS. Based on these intersection genes, we constructed a protein-protein interaction (PPI) network (**Figure 1E**), visually demonstrating the complex mutual regulatory relationships among targets. To further elucidate the mechanism of action, we performed functional enrichment analysis. GO analysis (**Figure 1F**) showed the distribution of targets in biological processes, cellular components, and molecular functions. KEGG pathway enrichment analysis (**Figure 1G**) further revealed key signaling pathways, showing significant enrichment in PI3K-Akt, MAPK, TNF, JAK-STAT, and Apoptosis signaling pathways. These pathways are widely involved in inflammatory responses, immune regulation, and apoptosis processes, suggesting that P. odoratum may exert therapeutic effects through multi-pathway synergistic mechanisms.

### 3.3 Identification of candidate target CCR1 based on transcriptomic data integration

To further narrow the candidate target range and improve screening accuracy, we integrated network pharmacology predictions with previously identified differentially expressed genes (DEGs). First, to ensure comprehensiveness of disease targets, we constructed a pSS disease target set by integrating data from GeneCards, OMIM, DisGeNET, and TTD databases (**Figure 1H**). Then, a PPI network was constructed based on predicted targets (**Figure 1I**), with topological analysis showing TNF, STAT3, HIF1A, and EGFR as hub nodes with high connectivity, indicating their important position in the regulatory network. Subsequently, we sought to find molecules that were both action targets of P. odoratum active components, played key pathogenic roles in pSS, and showed significant expression differences in clinical samples. To this end, we intersected “P. odoratum active component targets,” “pSS disease targets,” and “pSS differentially expressed genes (DEGs)” (**Figure 1J**). Venn diagram results showed only one gene simultaneously meeting all three conditions: chemokine receptor 1 (CCR1). This result identifies CCR1 as the top-ranked computational candidate target of P. odoratum warranting further investigation, thus we focused on it for subsequent mechanistic studies.

### 3.4 External dataset validation and correlation analysis of CCR1 with clinical features

**Figure 2:**
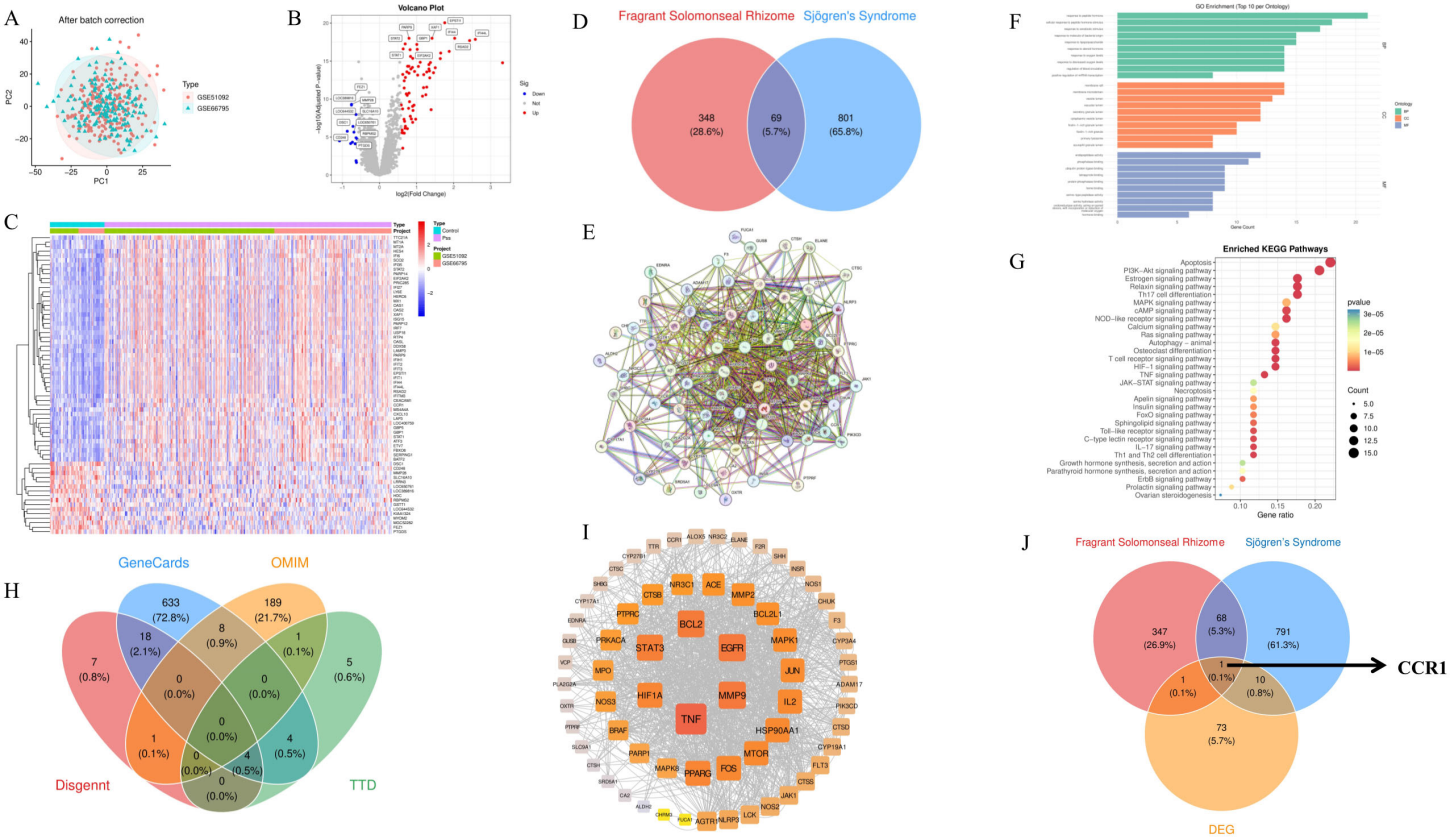
External validation, clinical correlation analysis, and GSEA enrichment analysis of CCR1. **(A)** Violin plot of CCR1 expression differences between pSS patients and healthy controls in external validation set (P = 0.00042); **(B)** ROC curve of CCR1 diagnostic performance, AUC = 0.758 (95% CI: 0.633–0.882); **(C–F)** Scatter plots of CCR1 expression level correlation with clinical indicators: IgG **(C)**, IgM **(D)**, IgA **(E)**, and ESSDAI score **(F, G)** Box plot of CCR1 expression level differences in analysis cohort (***P < 0.001); **(H)** ROC curve analysis of CCR1 in analysis cohort (AUC = 0.772, 95% CI: 0.712–0.828); **(I)** GSEA results showing significantly enriched KEGG pathways in CCR1 high expression group; **(J)** GSEA results showing significantly enriched KEGG pathways in CCR1 low expression group*.

To verify the reliability of the above findings, we introduced an external independent dataset (GSE84844) for validation. Consistent with discovery set results, CCR1 expression levels were significantly higher in pSS patients than healthy controls (P = 0.00042, **Figure 2A**). ROC curve analysis showed that CCR1 exhibited moderate discriminatory capacity in the external validation set, with AUC reaching 0.758 (95% CI: 0.633–0.882, **Figure 2B**), suggesting it warrants further evaluation as a candidate pSS biomarker. Furthermore, we evaluated the correlation between CCR1 expression and clinical immunological indicators and disease activity in pSS patients. Correlation analysis showed that CCR1 expression was significantly positively correlated with serum IgG levels (R = 0.49, P = 0.0057, **Figure 2C**), suggesting that CCR1 may be closely related to B cell hyperactivation and antibody production in pSS patients. However, in the samples included in this study, no statistically significant correlations were observed between CCR1 and IgM (R = 0.17, P = 0.36, **Figure 2D**), IgA (R = 0.045, P = 0.81, **Figure 2E**), or ESSDAI score (R = 0.21, P = 0.26, **Figure 2F**). Based on CCR1 expression levels, we also constructed a nomogram model for predicting pSS risk (**Supplementary Figure 1A**), with the calibration curve showing a mean absolute error (MAE) of only 0.019 (**Supplementary Figure 1B**), and both DCA and CIC demonstrating that the model provided significant net clinical benefit across a wide range of threshold probabilities (**Supplementary Figures 1C, D**).

### 3.5 GSEA analysis of CCR1-related potential biological functions and signaling pathways

To further reveal the specific biological functions of CCR1 in pSS pathogenesis, we divided samples into high and low expression groups based on CCR1 expression levels and performed gene set enrichment analysis (GSEA). First, we reconfirmed CCR1 expression differences and diagnostic value in this analysis cohort; results showed CCR1 was significantly highly expressed in the pSS group (P < 0.001, **Figure 2G**), with ROC curve AUC of 0.772 (95% CI: 0.712–0.828, **Figure 2H**), providing additional support for its candidacy as a biomarker warranting further validation. GSEA results showed that the CCR1 high expression group was significantly enriched in multiple signaling pathways closely related to immune inflammatory responses (**Figure 2I**), mainly including Chemokine signaling pathway, NOD-like receptor signaling pathway, RIG-I-like receptor signaling pathway, and Toll-like receptor signaling pathway. These results indicate from the transcriptomic level that CCR1 may mediate recruitment and infiltration of inflammatory cells by activating these innate immune pathways, thereby driving pSS pathological progression. Conversely, in the CCR1 low expression group (**Figure 2J**), enriched pathways mainly involved basic metabolism and cellular maintenance functions, such as Oxidative phosphorylation and Ribosome.

### 3.6 Immune infiltration landscape features and correlation analysis of CCR1 with immune microenvironment

**Figure 3:**
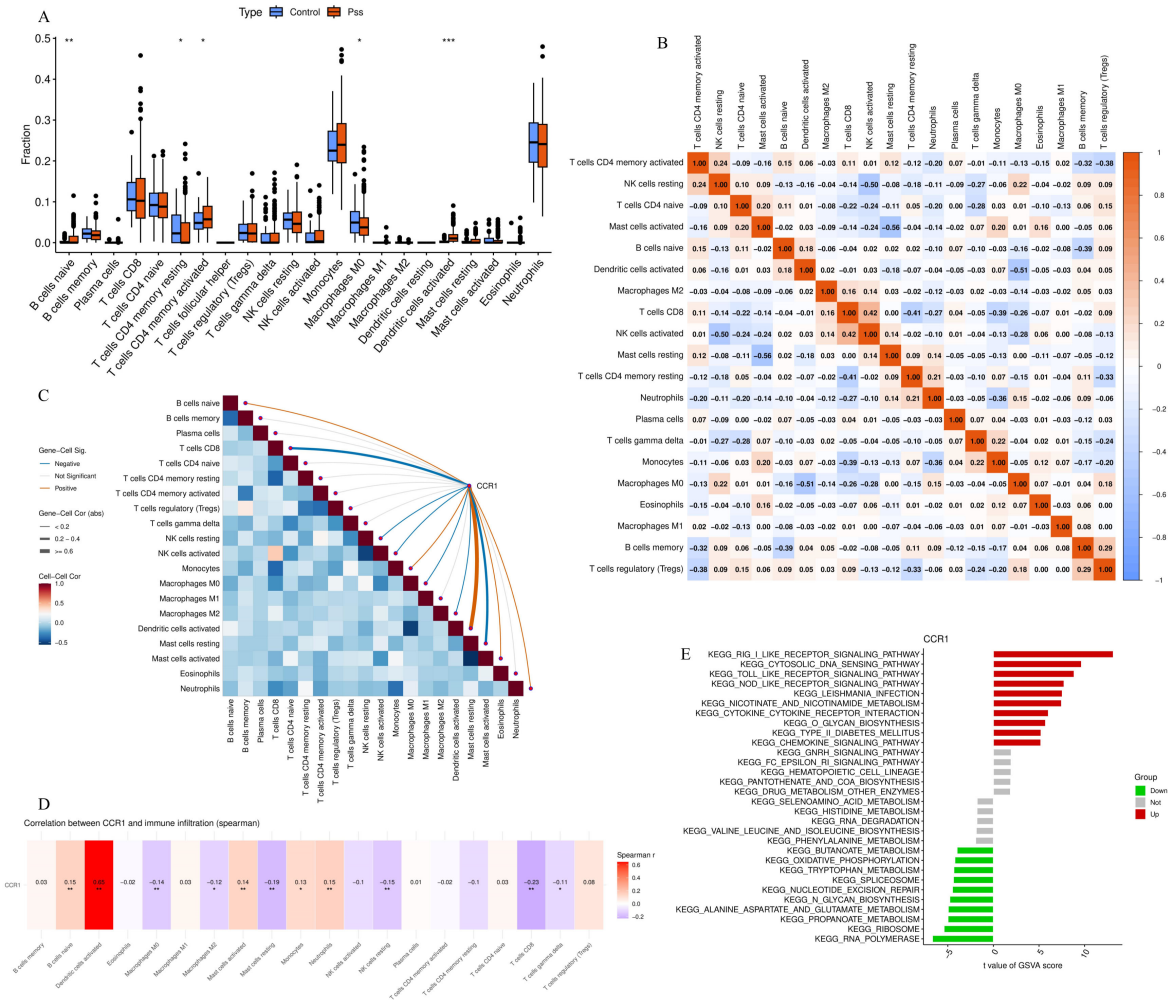
Immune infiltration features and correlation analysis of CCR1 with immune microenvironment. **(A)** Box plot of infiltration abundance of 22 immune cell types in pSS patients and healthy controls; **(B)** Correlation heatmap of 22 immune cell types; **(C)** Network visualization of immune cell interactions; **(D)** Spearman correlation analysis of CCR1 expression level with immune cell infiltration abundances; **(E)** GSVA-based pathway activity analysis. * P < 0.05; ** P < 0.01; *** P < 0.001.

To deeply explore pSS immune microenvironment characteristics, we used the CIBERSORT algorithm to quantify infiltration abundance of 22 immune cell types in pSS and control samples. Results showed (**Figure 3A**) that compared to healthy controls, the immune cell composition of pSS patients presented significant heterogeneity. Specifically, pro-inflammatory cells represented by M1 macrophages and activated CD4 memory T cells showed significantly elevated estimated infiltration levels in pSS peripheral blood samples, while anti-inflammatory cells such as regulatory T cells (Tregs) were relatively reduced. The correlation heatmap (**Figure 3B**) and network diagram (**Figure 3C**) of immune cells further revealed complex synergistic or antagonistic relationships among different immune cell subsets, constituting the characteristic disordered immune network of pSS. Subsequently, we focused on analyzing the correlation between candidate gene CCR1 and these immune cell infiltration abundances (**Figure 3D**). Spearman correlation analysis showed that CCR1 expression was most significantly positively correlated with monocytes (R = 0.65), suggesting that CCR1 may participate in regulating monocyte function. Additionally, CCR1 was negatively correlated with regulatory T cells (Tregs). GSVA analysis (**Figure 3E**) further validated these findings at the pathway level; CCR1 was significantly positively correlated with multiple innate immune recognition pathways including RIG-I-like receptor, Toll-like receptor, NOD-like receptor, and cytokine-receptor interaction pathways, while negatively correlated with metabolism-related pathways such as amino acid metabolism and ribosome. These results suggest that CCR1 expression is correlated with pro-inflammatory immune signatures in pSS peripheral blood, though the causal relationship remains to be established.

### 3.7 Validation of CCR1 expression in PBMCs of pSS patients

To understand the clinical characteristics of pSS patients in this study cohort, we compared baseline data between patient and healthy control groups (**Table 1**). Results showed that serum anti-SSA antibody (96.92%), anti-SSB antibody (76.92%), rheumatoid factor (103.01 ± 256.56 vs 11.16 ± 3.01 IU/mL, P = 0.015), erythrocyte sedimentation rate (66.06 ± 37.09 vs 10.86 ± 4.57 mm/h, P < 0.001), and C-reactive protein (23.32 ± 40.78 vs 3.81 ± 1.38 mg/L, P = 0.001) levels were significantly higher in pSS patients than healthy controls, with statistically significant differences. To further validate CCR1 expression in pSS, we used RT-qPCR to detect CCR1 mRNA relative expression levels in PBMCs from pSS patients (n = 65) and healthy controls (n = 48). As shown in **Supplementary Figure 2**, compared to the healthy control group, CCR1 mRNA expression was significantly elevated in PBMCs of pSS patients (P < 0.0001). These experimental results were consistent with bioinformatics analysis predictions, indicating at the clinical sample level that CCR1 is highly expressed in pSS patients, consistent with a potential association between CCR1 expression and pSS immunopathology, though functional validation is required.

**Table 1:** Comparison of clinical baseline characteristics between pSS patients and healthy controls.

Characteristics	Control (n=48)	PSS (n=65)	P-value
Age	56.56 ± 10.63	57.22 ± 10.48	0.746
Gender/Female	44 (91.67%)	61 (93.84%)	0.418
Alanine Aminotransferase (ALT) U/L	28.21 ± 55.91	22.89 ± 31.51	0.523
Aspartate Aminotransferase (AST) U/L	27.86 ± 23.32	24.51 ± 16.37	0.373
Alkaline Phosphatase U/L	83.14 ± 56.70	81.41 ± 40.57	0.851
γ-Glutamyl Transpeptidase U/L	40.78 ± 61.93	40.69 ± 44.26	0.992
Total Bilirubin umol/L	10.66 ± 5.62	9.75 ± 6.74	0.454
Uric Acid umol/L	268.67 ± 93.37	272.52 ± 73.49	0.816
Creatinine umol/L	79.78 ± 144.24	59.12 ± 16.67	0.269
Carbon Dioxide Combining Power mmol/L	22.45 ± 3.43	21.36 ± 2.91	0.082
Cholesterol mmol/L	4.54 ± 1.15	4.35 ± 0.90	0.337
Triglycerides mmol/L	1.37 ± 0.66	1.29 ± 0.56	0.516
High-Density Lipoprotein Cholesterol mmol/L	1.19 ± 0.35	1.08 ± 0.30	0.076
Low-Density Lipoprotein Cholesterol mmol/L	2.84 ± 0.92	2.79 ± 0.76	0.771
α-Hydroxybutyrate Dehydrogenase U/L	145.78 ± 33.75	140.27 ± 47.99	0.532
Creatine Kinase U/L	72.02 ± 62.05	79.88 ± 197.26	0.806
Lactate Dehydrogenase U/L	183.96 ± 45.50	181.99 ± 57.64	0.855
Potassium mmol/L	3.84 ± 0.38	3.96 ± 0.45	0.189
Sodium mmol/L	140.97 ± 2.83	140.37 ± 2.52	0.258
Chloride mmol/L	107.52 ± 3.58	106.97 ± 3.89	0.466
Calcium mmol/L	2.30 ± 0.15	2.25 ± 0.16	0.126
Magnesium mmol/L	0.93 ± 0.11	0.96 ± 0.11	0.216
Phosphorus mmol/L	1.17 ± 0.22	1.23 ± 0.18	0.199
Blood Glucose mmol/L	5.16 ± 1.15	4.86 ± 0.94	0.147
Erythrocyte Sedimentation Rate mm/h	10.86 ± 4.57	66.06 ± 37.09	<0.001
Anti-Streptolysin O IU/ml	60.51 ± 33.82	113.48 ± 95.05	<0.001
C-Reactive Protein (CRP) mg/L	3.81 ± 1.38	23.32 ± 40.78	0.001
Rheumatoid Factor (RF) IU/mL	11.16 ± 3.01	103.01 ± 256.56	0.015
Anti-SSA Antibody	0	63 (96.92%)	<0.001
Anti-SSB Antibody	0	50 (76.92%)	<0.001

### 3.8 Single-cell resolution CCR1 cell origin and expression localization

**Figure 4:**
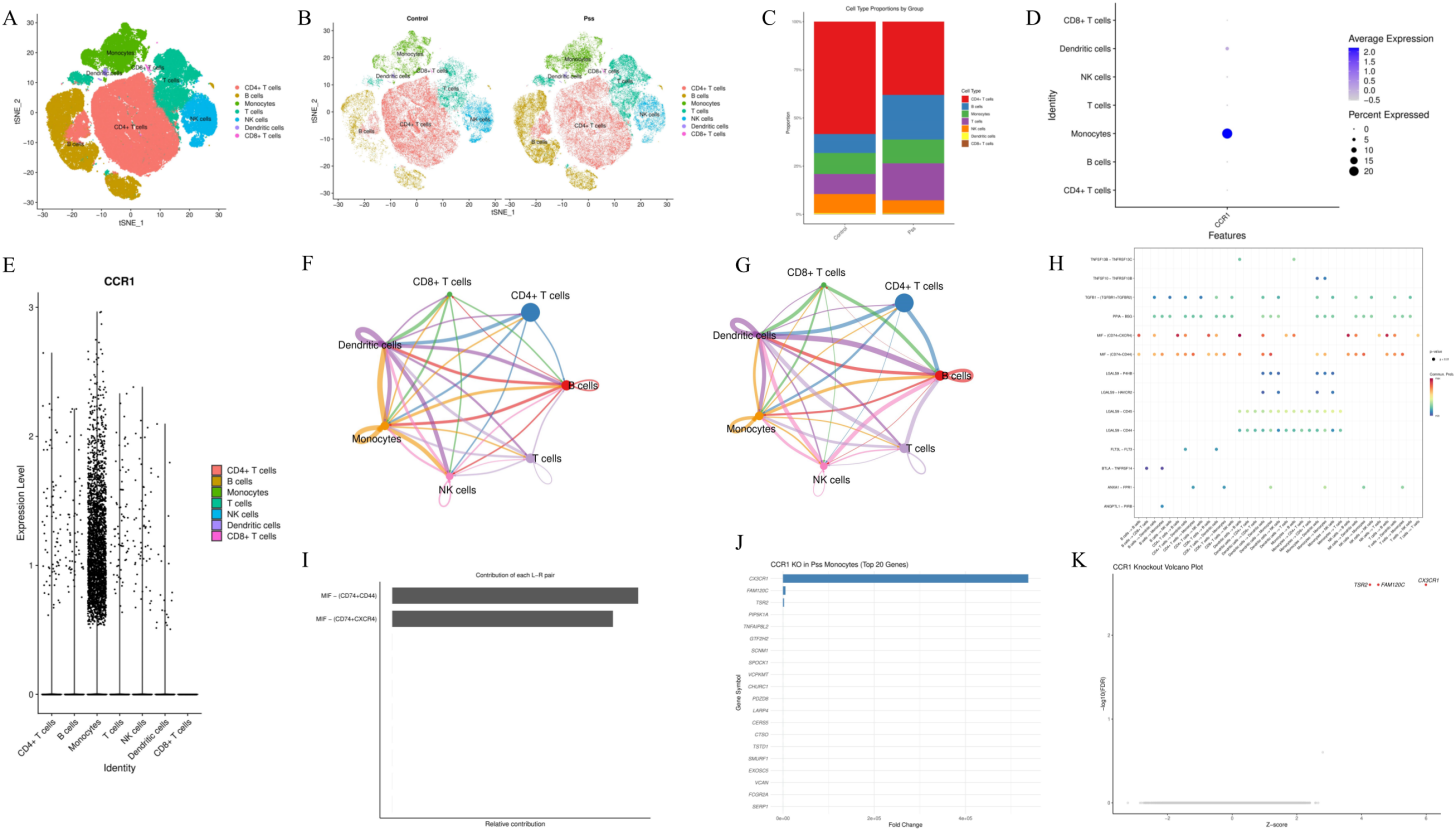
CCR1 expression localization analysis based on single-cell sequencing data, cell communication network, and virtual knockout analysis. **(A)** t-SNE clustering plot of all sample cell subpopulations; **(B)** t-SNE distribution plot distinguishing Control and Pss groups; **(C)** Stacked bar chart of relative proportions of each cell subpopulation in two groups; **(D)** Bubble plot of CCR1 expression in each cell subpopulation; **(E)** Violin plot of CCR1 expression levels in different cell subpopulations; **(F, G)** Cell-cell interaction network circle plots for Control **(F)** and Pss **(G)** groups; **(H)** Bubble plot of significantly enriched ligand-receptor pairs; **(I)** Relative contribution of ligand-receptor pairs in MIF signaling pathway; **(J)** Bar chart of top 20 genes with greatest expression change after CCR1 virtual knockout; **(K)** Volcano plot of differentially expressed genes after CCR1 virtual knockout.

To further clarify the specific cellular origin of CCR1 at single-cell level, we conducted in-depth mining of single-cell sequencing data from PBMCs of pSS patients and healthy controls (GSE253568, 9 pSS + 8 HC). Through t-SNE dimensionality reduction clustering analysis, we classified all cells into 7 major cell subpopulations (**Figure 4A**), including CD4+ T cells, CD8+ T cells, B cells, monocytes, NK cells, T cells, and dendritic cells, with each cell subpopulation distributed in both disease and control groups (**Figure 4B**). The stacked bar chart (**Figure 4C**) displayed the relative proportions of each cell subpopulation in the two groups. Subsequently, we focused on analyzing CCR1 expression abundance in these cell subpopulations. Bubble plot (**Figure 4D**) and corresponding violin plot (**Figure 4E**) results consistently showed that CCR1 exhibited a highly cell-specific expression pattern—CCR1 showed significant high expression in the monocyte population (~20% cells expressing, highest average expression), while it was almost undetectable or expressed at very low levels in lymphocyte populations including T cells, B cells, and NK cells. This finding indicates that CCR1 function in pSS is primarily associated with the monocyte subpopulation, suggesting that monocytes warrant further investigation as potential target cells in the context of CCR1-related immune processes.

### 3.9 Cell communication network overview and mif signaling pathway molecular mechanism

To comprehensively analyze cell communication characteristics in the pSS immune microenvironment, we used the CellChat algorithm to separately construct cell interaction networks for the control and pSS groups. The network circle plots showed (**Figures 4F, G**) that pSS group cell communication connections were more extensive and dense compared to controls, particularly with significantly enhanced signal interactions between monocytes and lymphocytes. Bubble plot analysis (**Figure 4H**) identified macrophage migration inhibitory factor (MIF) signaling pathway as one of the most active signaling pathways in the microenvironment, with MIF-(CD74+CD44) and MIF-(CD74+CXCR4) ligand-receptor pairs showing significant communication probability across multiple cell types. Ligand-receptor contribution analysis (**Figure 4I**) further showed that MIF signaling primarily depended on the MIF-(CD74+CD44) complex, with this pair’s relative contribution taking a dominant position, suggesting this may be the key molecular basis maintaining microenvironment immune homeostasis. Detailed CellChat comparisons including overall interaction number and strength changes, signaling pathway ranking, signal bubbles directed toward and from monocytes, outgoing/incoming signaling pattern heatmaps, and MIF pathway network circle plot are shown in **Supplementary Figures 5D–J**.

### 3.10 CCR1 virtual knockout analysis based on scTenifoldKnk

Given that single-cell analysis found CCR1 specifically highly expressed in monocytes, to further elucidate the specific regulatory role of CCR1 on monocyte biological functions, we used the scTenifoldKnk algorithm to construct a “Virtual Knockout” model of CCR1 in monocytes from single-cell datasets. Virtual knockout analysis results showed (**Figure 4J**) that CCR1 deletion caused significant remodeling of the monocyte transcriptome. The bar chart displayed the top 20 genes with greatest change magnitude, with CX3CR1 showing the greatest change, followed by FAM120C, TSR2, PIP5K1A, TNFAIP8L2, and others. The volcano plot (**Figure 4K**) further confirmed CX3CR1, TSR2, and FAM120C as the most significantly regulated genes by CCR1. Notably, CX3CR1 (another chemokine receptor) ranked among the genes with most significant changes, suggesting possible functional compensation or synergistic regulation between CCR1 and CX3CR1. Additionally, significant changes in VCAN (versican, extracellular matrix proteoglycan) and FCGR2A (Fc receptor IIA) indicate that CCR1 may play important roles in monocyte migration, extracellular matrix remodeling, and immune complex clearance. These results suggest from a computational perspective that CCR1 may occupy an important position in the predicted regulatory network of pSS monocytes.

### 3.11 Monocyte subtype characterization and CCR1 subtype-specific expression

**Figure 5:**
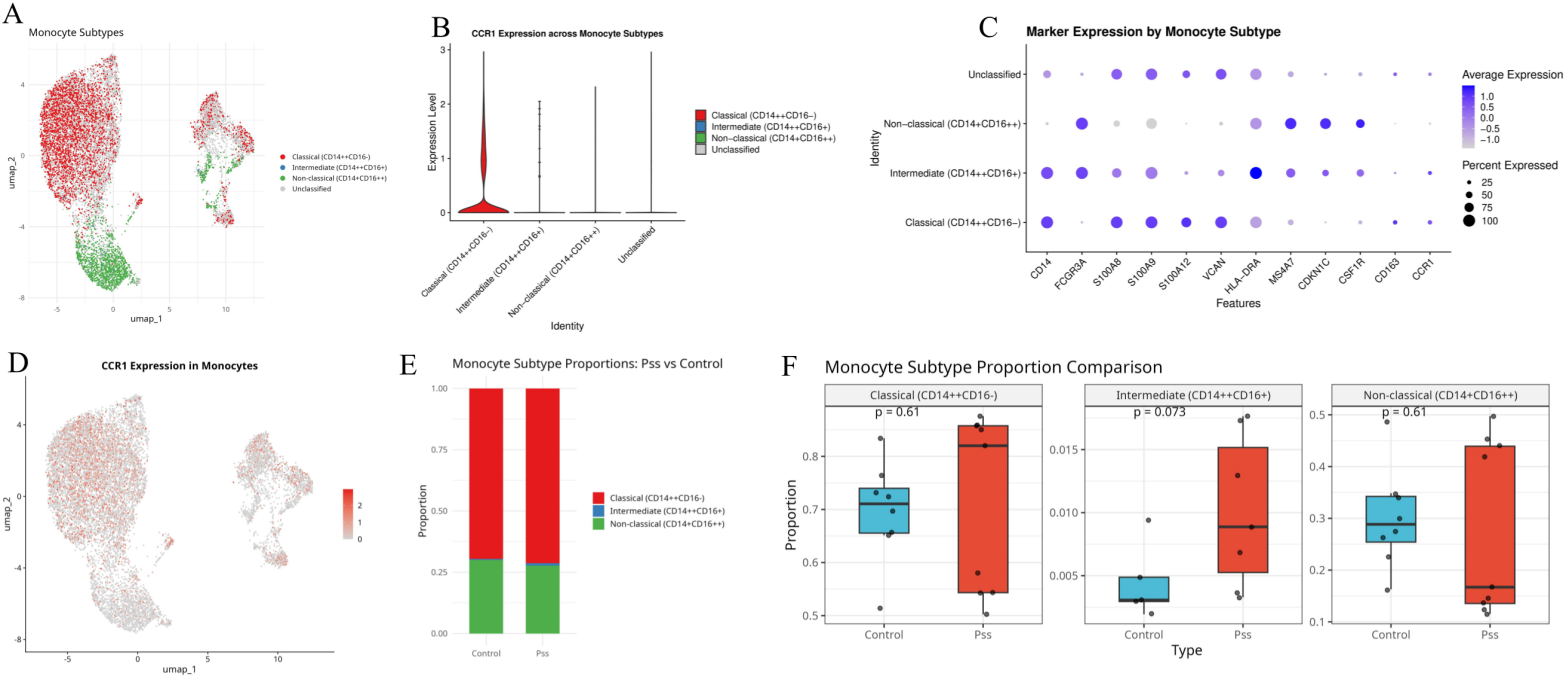
Monocyte subtype characterization and CCR1 expression profiling. **(A)** UMAP visualization of monocyte subtypes including classical (CD14++CD16−), intermediate (CD14++CD16+), non-classical (CD14+CD16++), and unclassified cells; **(B)** Violin plot of CCR1 expression across monocyte subtypes; **(C)** Bubble plot of monocyte subtype marker gene expression; **(D)** FeaturePlot of CCR1 expression on monocyte UMAP; **(E)** Stacked bar chart of monocyte subtype proportions comparing pSS and Control groups; **(F)** Box plot comparison of subtype proportions between groups with Wilcoxon test P values.

Having established that CCR1 was specifically highly expressed in monocytes, we further conducted sub-clustering analysis of monocytes to finely dissect the expression landscape of CCR1 across different monocyte functional subtypes. Through re-clustering and dimensionality reduction analysis of the monocyte population, combined with expression patterns of classic surface markers CD14 and FCGR3A (CD16), we classified monocytes into three functional subtypes (**Figure 5A**): classical monocytes (Classical, CD14++CD16−), intermediate monocytes (Intermediate, CD14++CD16+), and non-classical monocytes (Non-classical, CD14+CD16++), with a small number of unclassified cells. The bubble plot displayed the marker gene expression profile of each subtype (**Figure 5C**): classical monocytes highly expressed CD14, S100A8, S100A9, S100A12, and VCAN; intermediate monocytes highly expressed HLA-DRA, CSF1R, and CD163; non-classical monocytes highly expressed FCGR3A, MS4A7, and CDKN1C, consistent with previously reported monocyte classification standards. The detailed marker gene list for each sub-cluster is provided in **Supplementary Table 1**, and detailed information including sub-cluster UMAP, marker gene violin plots, bubble plots, and FeaturePlots during the clustering process are shown in **Supplementary Figures 3A–D**.

Subsequently, we focused on examining CCR1 expression differences across subtypes. Violin plot (**Figure 5B**) and FeaturePlot (**Figure 5D**) showed that CCR1 was predominantly highly expressed in classical monocytes, with partial expression in intermediate monocytes, while expression levels were low in non-classical monocytes. This finding suggests that CCR1 function may be closely related to the pro-inflammatory effects of classical monocytes. Further subtype proportion analysis showed (**Figure 5E**) that classical monocytes dominated in pSS patients. Proportion comparison box plots between pSS and control groups (**Figure 5F**) showed that classical (P = 0.61) and non-classical (P = 0.61) monocyte proportion differences did not reach statistical significance, and intermediate monocytes showed a non-significant increasing trend in pSS (P = 0.073). These proportion differences should be interpreted with caution given the limited sample size.

### 3.12 Disease-specific enrichment of CCR1-positive monocytes and milo differential abundance analysis

**Figure 6:**
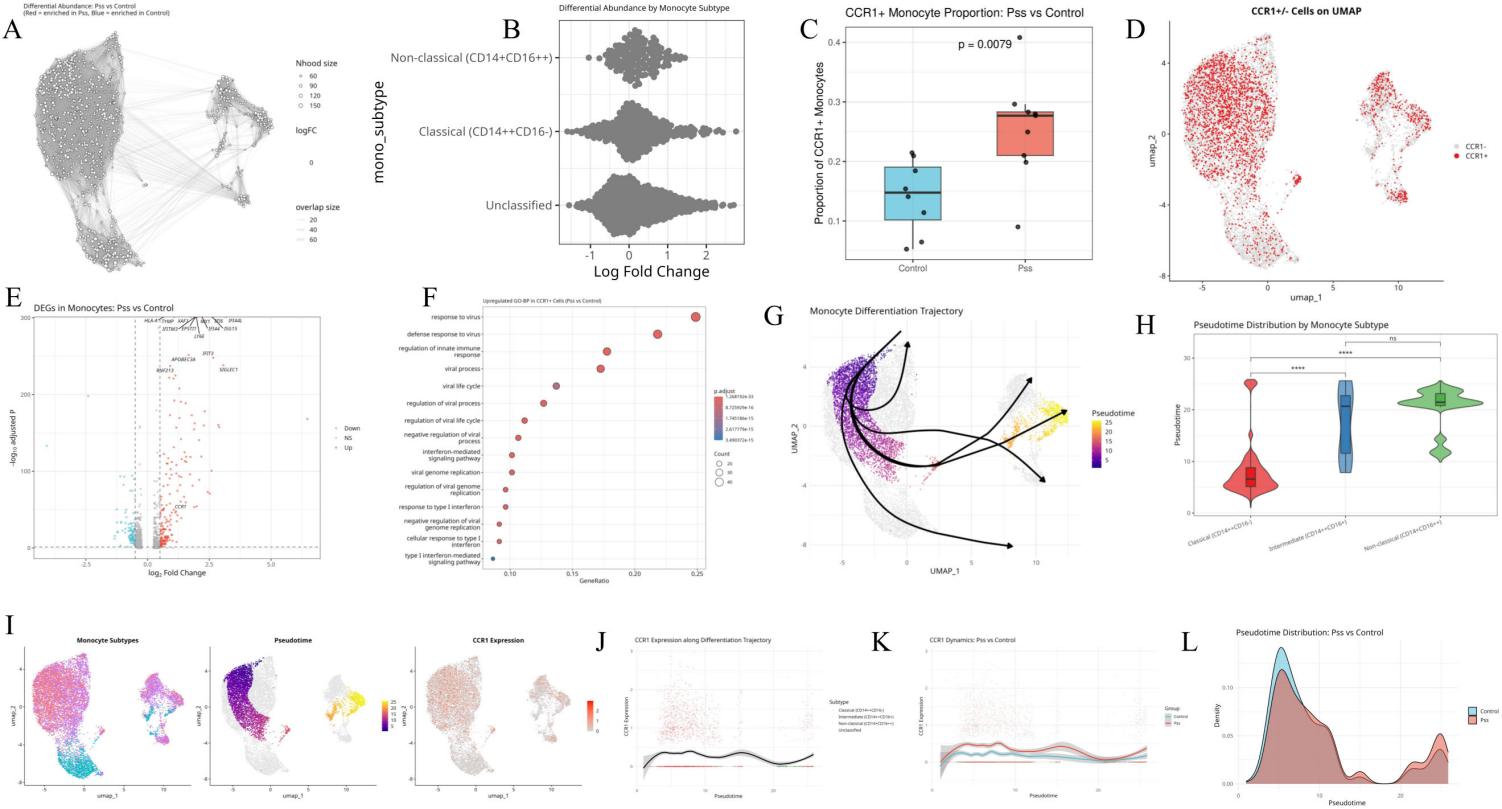
CCR1-positive monocyte disease-specific analysis, Milo differential abundance testing, and Slingshot pseudotime analysis. **(A)** Milo differential abundance UMAP, red nodes represent pSS-enriched neighborhoods, blue represents control-enriched neighborhoods; **(B)** Beeswarm plot of differential abundance by monocyte subtype; **(C)** Box plot comparing CCR1+ monocyte proportions between pSS and Control (P = 0.0079); **(D)** UMAP distribution of CCR1+/− cells; **(E)** Volcano plot of pSS vs Control monocyte DEGs; **(F)** GO-BP enrichment analysis of upregulated genes in CCR1+ monocytes; **(G)** Slingshot pseudotime trajectory plot, color represents differentiation time (Pseudotime), arrows indicate inferred differentiation directions; **(H)** Box plot of Pseudotime distribution by monocyte subtype (****P < 0.0001; ns = not significant (P ≥ 0.05)); **(I)** Triple comparison panels: subtype, pseudotime, and CCR1 expression; **(J)** CCR1 expression dynamics along pseudotime trajectory; **(K)** CCR1 pseudotime dynamics comparing pSS and Control; **(L)** Pseudotime density distribution plot showing differentiation bias between pSS and Control.

To evaluate the differential abundance of monocyte neighborhoods in pSS without pre-defined cluster assignments, we employed the Milo algorithm for differential abundance analysis. Milo analysis results showed (**Figure 6A**) that on the UMAP, nodes of different sizes and colors displayed the enrichment direction of each cell neighborhood, with red nodes representing neighborhoods enriched in pSS and blue nodes representing those enriched in controls. The beeswarm plot (**Figure 6B**) displayed the log fold change distribution of each neighborhood by monocyte subtype, showing that both classical and non-classical monocyte neighborhoods exhibited non-significant trends toward pSS enrichment (minimum SpatialFDR = 0.107, not reaching the conventional significance threshold). Among all 996 detected neighborhoods, 589 (59.1%) showed positive logFC values (i.e., higher abundance in pSS); detailed results are provided in **Supplementary Table 2**.

Next, we defined monocytes with CCR1 expression > 0 as CCR1-positive (CCR1+) cells and analyzed their enrichment in pSS. Box plot results showed (**Figure 6C**) that the proportion of CCR1+ monocytes among total monocytes was significantly higher in pSS patients than healthy controls (P = 0.0079), clearly demonstrating that CCR1+ monocytes undergo disease-specific expansion in pSS. The UMAP distribution plot (**Figure 6D**) intuitively showed the spatial distribution of CCR1-positive and negative cells in the dimensionality reduction space, with CCR1+ cells (red) primarily clustered in classical monocyte regions. The split UMAP of CCR1+ cells in pSS vs Control is shown in **Supplementary Figure 3E**.

To reveal the transcriptomic characteristics of pSS monocytes, we performed differential expression analysis between pSS and control monocytes. The volcano plot (**Figure 6E**) showed that pSS monocytes had 183 significantly upregulated and 97 significantly downregulated genes (**Supplementary Table 3**), with interferon-stimulated genes including IFI44L, IFI6, and ISG15 ranking among the top upregulated genes. CCR1 was also among the significantly upregulated genes (log2FC = 1.15, adj.P = 2.79 × 10^-48^). Furthermore, we performed GO-BP enrichment analysis on upregulated genes in CCR1+ monocytes compared to CCR1− monocytes in the pSS group (**Supplementary Table 4**; **Figure 6F**), showing significant enrichment in response to virus, regulation of innate immune response, and type I interferon mediated signaling pathway. Additionally, KEGG pathway enrichment results of CCR1+ monocyte upregulated genes (**Supplementary Figure 3F**) showed significant enrichment in coronavirus disease, influenza A, Epstein-Barr virus infection, phagosome, NOD-like receptor signaling pathway, and antigen processing and presentation. These results indicate that CCR1+ monocytes in pSS display functional characteristics of enhanced innate immune responses and activated antiviral responses.

### 3.13 Slingshot pseudotime analysis reveals monocyte differentiation bias and CCR1 expression dynamics in pSS

Having established that CCR1 was specifically enriched in classical monocytes and significantly expanded in pSS, we further employed the Slingshot algorithm for pseudotime analysis of monocytes to reveal the dynamic changes in CCR1 expression along the monocyte differentiation trajectory. Using classical monocytes (representing the most primitive state) as the starting point, Slingshot successfully reconstructed continuous differentiation trajectories from classical to intermediate to non-classical monocytes (**Figure 6G**), with trajectories marked by arrows on the UMAP. The distribution of subtypes along the pseudotime axis (**Figure 6H**) showed that classical monocytes had the lowest Pseudotime values (early differentiation), non-classical had the highest (late differentiation), with intermediate in between, and inter-subtype differences were highly significant (P < 0.0001), validating the biological plausibility of trajectory reconstruction. The triple comparison panels (**Figure 6I**) visualized subtype distribution, Pseudotime gradient, and CCR1 expression intensity in the same dimensionality reduction space, intuitively showing that CCR1 was mainly highly expressed in classical monocytes at the early differentiation stage.

CCR1 expression dynamics along Pseudotime (**Figure 6J**) revealed an important finding: CCR1 maintained relatively high expression levels during the early differentiation stage and gradually decreased as monocytes differentiated toward the non-classical direction. Group comparison (**Figure 6K**) showed that CCR1 expression in pSS patients was higher than controls across Pseudotime stages. The Pseudotime density distribution plot (**Figure 6L**) further revealed differentiation bias between pSS and controls—the Pseudotime distribution of pSS monocytes was overall shifted toward higher values, suggesting a computational inference of possible differences in monocyte differentiation dynamics between pSS and controls, though pseudotime ordering does not constitute direct evidence of *in vivo* differentiation trajectories.Additionally, CCR1 expression analysis by differentiation stage (Stage 1–5) (**Supplementary Figures 4C, D**) showed that CCR1 in the pSS group was significantly higher than controls at Stage 1–2 (****, P < 0.0001) and Stage 3–4 (***, P < 0.001), with expression approaching silencing in Stage 5. Stage proportion changes between pSS and controls (**Supplementary Figure 4E**) showed increasing proportions in mid-to-late stages (Stage 3–5) in the pSS group. Multi-gene expression dynamics along pseudotime and group comparisons are detailed in **Supplementary Figures 4A, B**. CCR1 expression across all cell types in the pSS vs Control split bubble plot is shown in **Supplementary Figure 4F**.

### 3.14 SCENIC predicts candidate upstream transcription factors associated with CCR1

**Figure 7:**
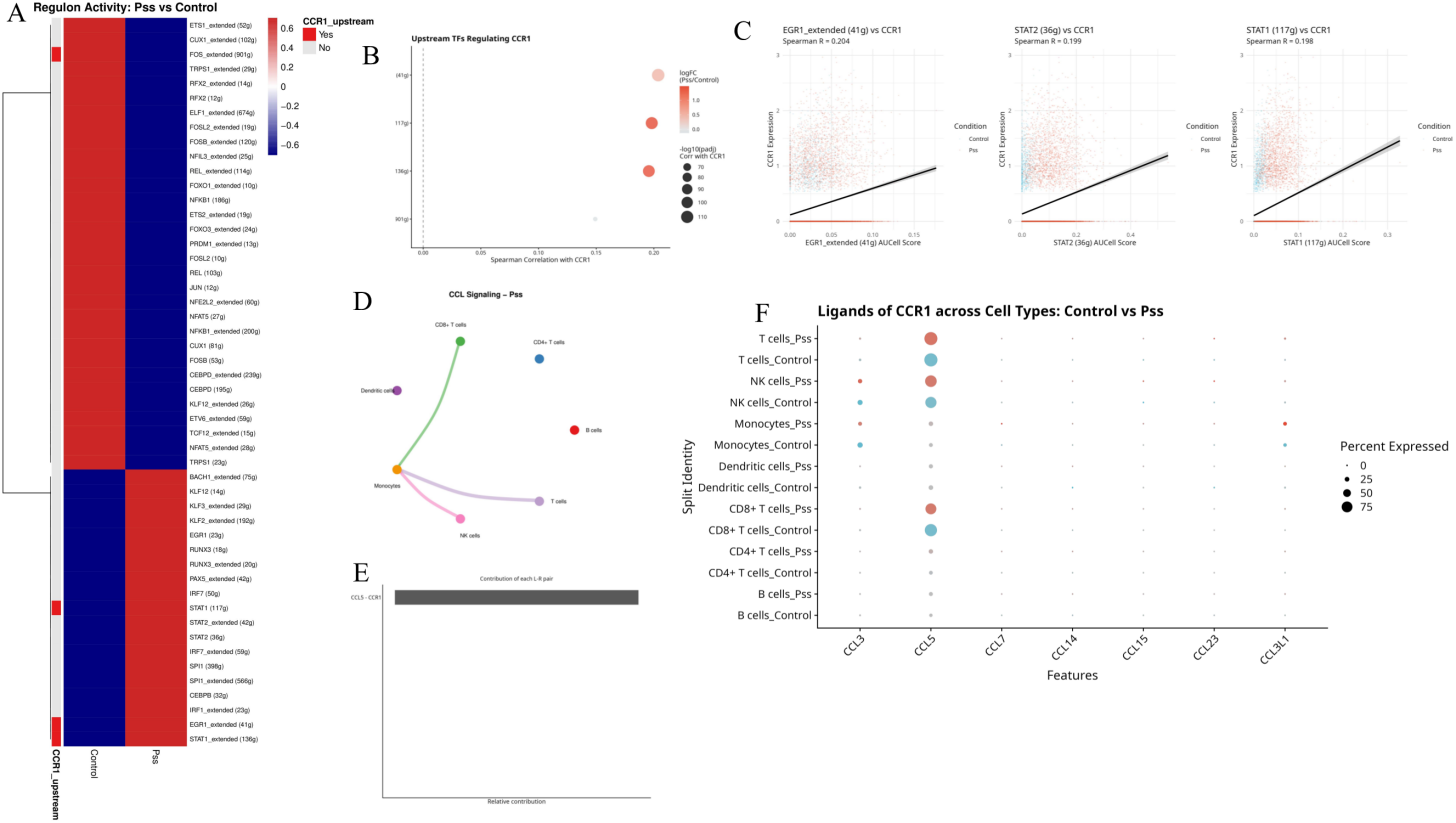
SCENIC transcription factor regulatory network analysis and CCL5–CCR1 chemotactic axis cell communication analysis. **(A)** Regulon activity heatmap between pSS and Control, left-side red marks (CCR1_upstream) annotate upstream transcription factors directly targeting CCR1; **(B)** Integrated bubble plot of upstream key transcription factors, x-axis represents correlation with CCR1, y-axis/color represents pSS vs Control logFC, bubble size represents statistical significance; **(C)** Scatter correlation plots of top 3 transcription factors (EGR1_extended, STAT2, STAT1) with CCR1 expression levels; **(D)** CCL signaling pathway network circle plot in pSS group, showing CCL5–CCR1 chemotactic axis network structure; **(E)** Relative contribution of ligand-receptor pairs in CCL pathway, CCL5-CCR1 dominates; **(F)** Expression bubble plot of CCR1 ligands (CCL3, CCL5, CCL7, etc.) across cell types comparing pSS vs Control.

To reveal the transcriptional regulatory mechanisms driving CCR1 aberrant upregulation, we used the SCENIC algorithm to construct a transcription factor regulatory network (Regulon) in monocytes. Based on Regulon activity scores, we compared Regulon activity differences between pSS and control groups (**Figure 7A**). The heatmap showed extensive Regulon activity changes in the pSS group (detailed in **Supplementary Table 7**). Particularly, we used CCR1_upstream annotations (red marks on the left side of **Figure 7A**) to identify transcription factors identified by SCENIC analysis as predicted to target the CCR1 promoter region, including STAT1, EGR1_extended, FOS_extended, and STAT1_extended (**Supplementary Table 5**).To further quantify the key characteristics of these upstream transcription factors, we generated an integrated bubble plot (**Figure 7B**) simultaneously incorporating three dimensions of information: Spearman correlation with CCR1 (x-axis), pSS vs Control logFC (y-axis/color), and statistical significance of the Regulon (bubble size). Results showed that EGR1_extended (41 target genes, R = 0.204), STAT2 (36 target genes, R = 0.199), and STAT1 (117 target genes, R = 0.198) were the transcription factors with strongest correlation with CCR1 expression and significant activation in pSS (**Supplementary Table 6**). Scatter plots (**Figure 7C**) intuitively displayed the positive correlations between AUCell scores of EGR1_extended, STAT2, and STAT1 and CCR1 expression levels. Of particular note, STAT1 and STAT2 showed substantial activity upregulation in pSS (STAT1 logFC = 1.48, STAT2 logFC = 2.04), while IRF7, though with slightly lower CCR1 correlation (R = 0.165), exhibited the highest activation fold-change in pSS (logFC = 2.34), suggesting the STAT-IRF axis may be a candidate regulatory pathway driving CCR1 transcriptional upregulation in pSS monocytes (**Supplementary Table 8**). Regulon activity heatmap by monocyte subtype, key transcription factor UMAP activity distributions, and pSS vs Control violin plot comparisons are detailed in **Supplementary Figures 5A–C**.

### 3.15 CCL5–CCR1 chemotactic axis cell communication analysis

Having established the specific high expression of CCR1 in monocytes and identified upstream transcription factor regulation, we further focused on the CCR1-related chemokine signaling network to explore how CCR1 mediates cell communication in the pSS immune microenvironment through ligand-receptor interactions. Using the CellChat algorithm, we constructed a cell communication network for the CCL signaling pathway in the pSS group. The CCL pathway circle plot (**Figure 7D**) clearly displayed the directionality of CCL signaling among immune cell subpopulations, with T cells and NK cells as the main signal senders and monocytes and dendritic cells as the main signal receivers. Ligand-receptor contribution analysis (**Figure 7E**) showed that the CCL5-CCR1 pair dominated the entire CCL signaling pathway, revealing the predicted signaling axis of “T/NK cell-secreted CCL5 engaging CCR1 to mediate monocyte chemotactic recruitment.” Furthermore, we systematically analyzed the expression levels and pSS vs Control changes of all known CCR1 ligands (CCL3, CCL5, CCL7, CCL14, CCL15, CCL23, CCL3L1) across cell types (**Figure 7F**). Results showed that CCL5 was significantly upregulated in T cells and NK cells in the pSS group, CCL3 also showed some degree of upregulation in T cells and NK cells, while other ligands such as CCL7 had relatively low expression levels in peripheral blood cells. Violin plot comparisons of CCR1 ligands in monocytes between pSS and Control and the complete expression profile of CCL pathway genes are detailed in **Supplementary Figures 4G, H**. These results collectively suggest a predicted chemotactic signaling axis: in pSS patients, T/NK cells promote monocyte chemotactic recruitment and inflammatory aggregation through upregulating secretion of CCL5/CCL3 and other chemokines, mediated via the CCR1 receptor, potentially contributing to the immunopathological processes of the disease, pending experimental confirmation.

### 3.16 Construction of ceRNA regulatory network targeting CCR1 and identification of key upstream lncRNAs

**Figure 8:**
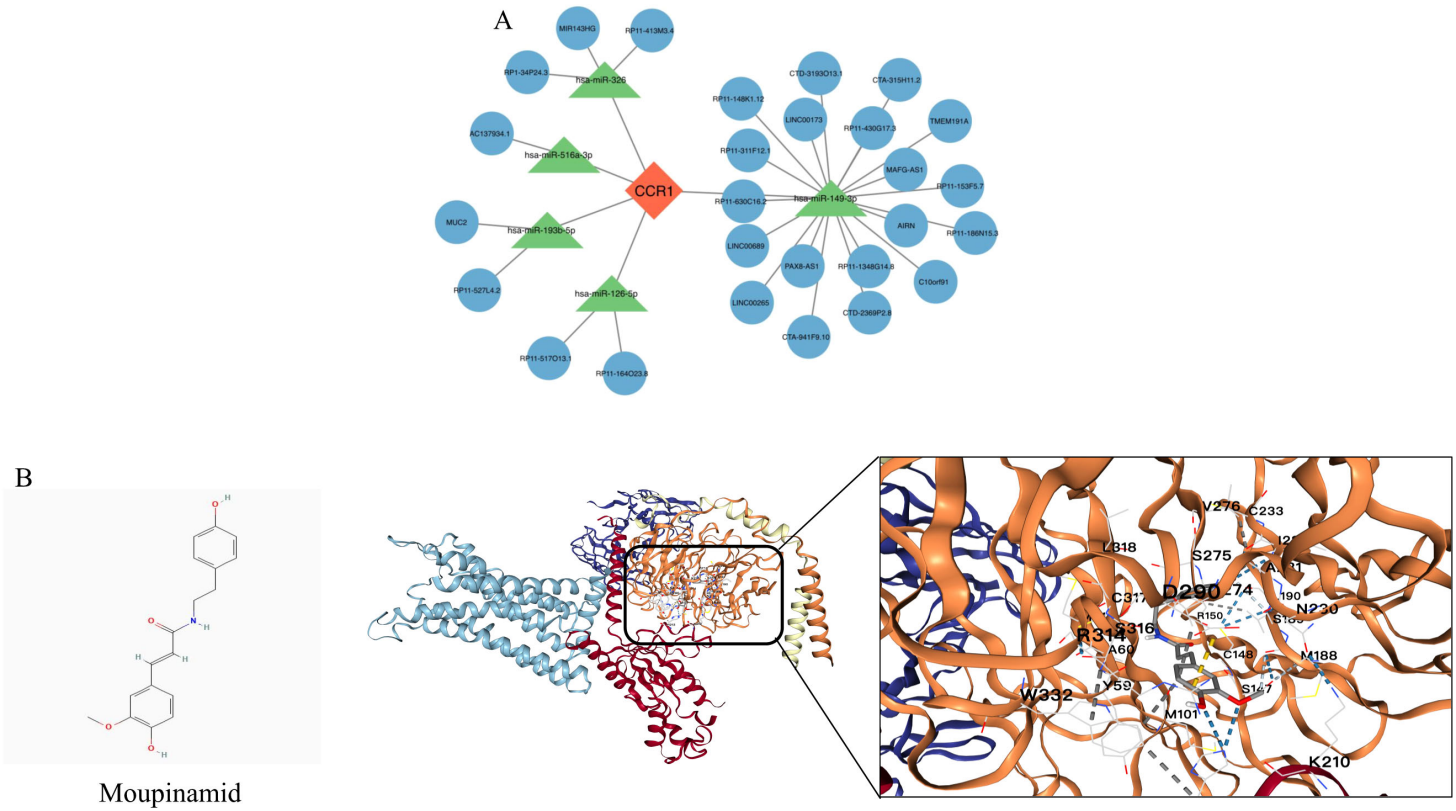
ceRNA regulatory network targeting CCR1 and molecular docking validation. **(A)** lncRNA-miRNA-mRNA ceRNA regulatory network, red diamond node represents CCR1, green triangle nodes represent key miRNAs, blue circle nodes represent upstream lncRNAs; **(B)** Molecular docking results of (P) odoratum active component Moupinamide with CCR1 protein: left shows Moupinamide molecular structure, center shows 3D complex overview, right shows detailed local interaction diagram of binding site.

To deeply analyze the post-transcriptional regulatory mechanisms leading to abnormal high expression of CCR1 in pSS monocytes, we constructed a lncRNA-miRNA-mRNA competing endogenous RNA (ceRNA) regulatory network centered on CCR1 based on multi-database joint prediction (**Figure 8A**). Network topology analysis showed that this regulatory axis contained 33 nodes, with CCR1 as the central regulated mRNA, directly negatively regulated by 5 key miRNAs including hsa-miR-149-3p, hsa-miR-326, hsa-miR-516a-3p, hsa-miR-193b-5p, and hsa-miR-126-5p. Among these miRNAs, hsa-miR-149-3p showed the highest connectivity, serving as the central hub node connecting CCR1 with upstream lncRNA populations. Further analysis found that 27 lncRNAs including LINC00173, MAFG-AS1, and PAX8-AS1 had confirmed targeted binding relationships with hsa-miR-149-3p. These results suggest that these upstream lncRNAs may competitively adsorb hsa-miR-149-3p by serving as “miRNA sponges,” thereby relieving the post-transcriptional inhibition of CCR1 by this miRNA, ultimately driving aberrant upregulation of CCR1 expression levels.

### 3.17 Molecular docking validation of core component moupinamide with CCR1

To explore the potential binding interaction between P. odoratum active components and CCR1 at the molecular level, we performed molecular docking simulation between the representative component Moupinamide and CCR1 protein (**Figure 8B**). Based on receptor protein surface features, we screened the optimal active pocket as the docking site, ensuring spatial rationality of ligand binding. Docking results showed that Moupinamide (MW = 313.35 Da, LogP = 2.39, HBD = 3, HBA = 4, TPSA = 78.79 Å², Lipinski violations = 0) could precisely embed deep into the CCR1 active pocket, with the lowest binding energy of −8.1 kcal/mol, suggesting predicted binding affinity in the computational model (below the −7.0 kcal/mol threshold for strong binding activity). Detailed binding mode analysis showed that the Moupinamide molecule adapted highly to the pocket microenvironment through conformational adjustment, with its polar groups forming multiple stable hydrogen bonds with key amino acid residues ASP-290, ARG-314, SER-316, and MET-101, while also producing hydrophobic interactions with residues TYR-59, MET-188, and VAL-276. This predicted binding mode, dominated by hydrogen bond networks and assisted by hydrophobic interactions, provides a computational rationale suggesting how Moupinamide may interact with CCR1, warranting further experimental validation.Notably, the binding site of Moupinamide overlaps substantially with the predicted chemokine ligand binding interface. Spatially, the docking pose reveals that Moupinamide forms key interactions with residues distributed across transmembrane helices TM3, TM5, TM6, and TM7, collectively constraining the relative positions of these helices. In particular, these interactions are predicted to prevent the outward movement of TM6, a conformational rearrangement essential for G-protein coupling in class A GPCRs. This mechanism is analogous to those observed in crystallographic studies of other CC chemokine receptor–small molecule complexes, such as CCR5 bound to Maraviroc and CCR2 bound to orthosteric antagonists, where ligand occupancy within the transmembrane pocket locks the receptor in an inactive state. Furthermore, several key interacting residues—particularly ASP-290 on TM7—are known to participate in the GPCR activation switch mechanism of CC chemokine receptors. Taken together, these structural observations suggest that Moupinamide binding may competitively block CCL5 access to the CRS2 (transmembrane pocket) region of CCR1 and constrain the receptor in an inactive conformation, with implications for both ligand-induced and constitutive receptor signaling (see Discussion for detailed mechanistic analysis).

## 4 Discussion

Primary Sjögren’s syndrome (pSS) is a systemic autoimmune disease with complex pathogenesis and strong clinical heterogeneity, currently lacking specific treatment targeting its etiology (26, 27). This study computationally prioritized CCR1 as a candidate target of P. odoratum for potential relevance to pSS through integrating transcriptomics, network pharmacology, single-cell sequencing, and clinical sample validation, and further elucidated its role in pSS monocyte immunopathology through subtype characterization, pseudotime analysis, SCENIC transcription factor networks, and CCL pathway communication analysis. The following discussion progressively elaborates on the candidate role of CCR1 in pSS from seven aspects: differential expression validation, cellular localization, subtype characterization, differentiation dynamics, transcriptional regulation, chemotactic signaling, and drug intervention.

A central observation of this study is the consistent upregulation of CCR1 in peripheral blood of pSS patients across multiple independent data sources: the training set (GSE51092 + GSE66795) showed significantly high CCR1 expression in the pSS group; the independent validation set (GSE84844) further confirmed this trend (P = 0.00042, AUC = 0.758); clinical sample RT-qPCR testing ultimately confirmed CCR1 upregulation at the mRNA level (P < 0.0001). The concordance across these independent analyses suggests that CCR1 upregulation is a reproducible molecular feature of pSS, though its functional significance requires experimental validation. Additionally, CCR1 was significantly positively correlated with serum IgG levels (R = 0.49, P = 0.0057), and since elevated IgG is a marker of humoral immune hyperactivation in pSS, this suggests a potential functional link between CCR1 and B cell-mediated immune dysregulation. It should be noted that no significant correlation was observed between CCR1 and ESSDAI score (R = 0.21, P = 0.26), which may be related to limited sample size or the multidimensional nature of ESSDAI assessment. This raises a critical question: in which cell type does CCR1 exert its function? This prompted us to conduct cellular localization analysis.

Single-cell sequencing analysis provided a precise answer. Among 7 major immune cell subpopulations, CCR1 was specifically highly expressed in monocytes (~20% expressing cells, highest average expression), with barely detectable expression in T cells, B cells, and NK cells. This finding is consistent with previous reports that CCR1, as a CC chemokine receptor family member, is primarily expressed on myeloid cells (28, 29), and precisely anchored the potential function of CCR1 in pSS to monocytes. Recent studies have highlighted that peripheral blood monocytes play a critical bridging role in multiple autoimmune diseases, connecting innate and adaptive immune responses (30), and our findings provide new molecular-level support for this concept in pSS. Having established cellular localization, a natural question arises: within the functionally heterogeneous monocyte population, which functional subtype is CCR1 enriched in?

To address this question, we further classified monocytes into three functional subtypes: classical (CD14++CD16−), intermediate (CD14++CD16+), and non-classical (CD14+CD16++), consistent with the internationally recognized monocyte classification standard (31). Analysis revealed that CCR1 was predominantly expressed in classical monocytes. This finding carries important functional implications—classical monocytes are the most abundant peripheral blood monocyte subtype (typically 80%–90%), possessing robust phagocytic activity and pro-inflammatory cytokine release capacity, playing a central role in early infection and inflammation responses (32). The predominant expression of CCR1 in this subtype suggests that CCR1-mediated chemotactic recruitment in pSS primarily relies on this highly pro-inflammatory cell population. Furthermore, CCR1-positive monocytes were significantly expanded in pSS (P = 0.0079), further confirming the tight association between CCR1 and disease activity. Although subtype proportions did not reach statistical significance between pSS and controls (classical P = 0.61, non-classical P = 0.61), intermediate monocytes showed an increasing trend (P = 0.073), consistent with previously reported intermediate monocyte expansion in inflammatory diseases (33). Limited sample size may be a contributing factor to the insufficient statistical power.

Having established CCR1’s predominant expression in classical monocytes, we sought to further understand the biological significance of this expression pattern from the perspective of differentiation dynamics. Using the Slingshot algorithm, we reconstructed continuous differentiation trajectories from classical to intermediate to non-classical monocytes and analyzed CCR1 expression dynamics along the differentiation axis—a unique highlight distinguishing this study from similar work. Results showed that CCR1 maintained high expression during early differentiation (classical stage) and gradually decreased along the differentiation progression, a dynamic pattern providing temporal corroboration of the static observation of CCR1 predominance in classical monocytes. More importantly, pseudotime density distributions differed markedly between pSS patients and healthy controls, suggesting that the monocyte differentiation process undergoes systemic remodeling in pSS. This finding provides a new dimension for understanding monocyte functional abnormalities in pSS—not merely the upregulation of a single gene, but systematic changes in differentiation dynamics that may collectively drive disease immunopathology. It should be noted that pseudotime analysis is a computational inference method, and its conclusions require further validation through experimental approaches such as lineage tracing (21).

What are the molecular mechanisms underlying CCR1 upregulation in pSS monocytes? To address this core question, we employed the SCENIC algorithm to construct a monocyte transcription factor regulatory network. The analysis identified STAT1 and EGR1 as transcription factors predicted to target the CCR1 promoter region. STAT1 is the core effector of the JAK-STAT signaling pathway and a key mediator of type I interferon responses (34). The STAT1 regulon was positively correlated with CCR1 expression (R = 0.198) and significantly upregulated in pSS (logFC = 1.48). Additionally, IRF7, while not a direct upstream TF, showed positive correlation with CCR1 (R = 0.165) and the highest activation amplitude in pSS (logFC = 2.34). Integrating these findings with the known core event of type I interferon signature activation in pSS (6), we can infer a potential regulatory pathway: type I interferon signal activation leading to STAT1/IRF7 regulon upregulation, subsequently driving CCR1 transcriptional enhancement and ultimately potentiating monocyte chemotactic function. We should candidly note that the correlation coefficients between regulons and CCR1 were generally modest (R ≈ 0.16–0.20), partly due to the inherent sparsity and noise of single-cell data, and partly reflecting the complex nature of CCR1 expression being co-regulated by multiple transcription factors rather than dominated by a single factor. These transcriptional regulatory relationships await further validation by ChIP-seq or luciferase reporter assays.

Having elucidated the upstream regulatory mechanisms of CCR1, the next question to address is: what role does CCR1 play in the intercellular communication network of the pSS immune microenvironment? CellChat analysis provided critical insights. In the CCL signaling pathway of the pSS group, CCL5-CCR1 was identified as the absolutely dominant ligand-receptor pair, with T cells and NK cells as the main sources of CCL5, recruiting monocytes to inflammatory sites through the CCL5–CCR1 signaling axis. CCL5 (RANTES) has been previously reported to be elevated in salivary gland tissue and saliva of pSS patients (35). This study provides computational single-cell level evidence consistent with the predicted activity of the CCL5–CCR1 signaling axis in pSS peripheral blood immune circulation. Integrating all preceding findings, we can construct a hypothetical mechanistic framework: in pSS, type I interferon signaling upregulates CCR1 transcription in classical monocytes through STAT1/IRF7; simultaneously, T/NK cells upregulate CCL5/CCL3 secretion; the CCL5–CCR1 chemotactic axis recruits CCR1-high classical monocytes to inflammatory sites, where they form positive feedback loops with dendritic cells and B cells through MIF and other pro-inflammatory signaling networks (36, 37), collectively maintaining and amplifying the chronic inflammatory state of pSS.

This mechanistic picture provides clear targets for therapeutic exploration. Through “drug target–disease gene–differential expression” three-dimensional intersection analysis, CCR1 was identified as the only candidate target simultaneously meeting all three conditions, offering a computational hypothesis consistent with the traditional “yin-nourishing and dryness-moistening” efficacy of P. odoratum from a network pharmacology perspective (38). Molecular docking predicted that Moupinamide may have binding affinity with CCR1 (−8.1 kcal/mol), achieving stable binding through hydrogen bonds with key residues ASP-290, ARG-314, and SER-316. Importantly, structural analysis of the docking complex provided further mechanistic insights. The binding site of Moupinamide overlaps with the predicted CCL5 binding interface within the orthosteric transmembrane pocket, suggesting competitive blockade of chemokine-induced receptor activation. The interaction with ASP-290 on TM7, a residue critically involved in the GPCR activation switch mechanism, suggests that Moupinamide may constrain CCR1 in an inactive conformation. Furthermore, given the well-documented constitutive (ligand-independent) activity of CCR1, Moupinamide may function as an inverse agonist, reducing basal receptor signaling below the constitutive baseline, although this remains speculative and requires pharmacological verification through GTPγS binding and β-arrestin recruitment assays. This potential dual-mechanism profile—simultaneously blocking CCL5-driven chemotaxis and reducing constitutive monocyte migration—would be highly relevant in pSS, where our single-cell analysis demonstrated both upregulated CCL5–CCR1 signaling and expanded CCR1-positive classical monocyte populations. Based on these computational findings, we propose the exploratory hypothesis that P. odoratum may interact with CCR1 through Moupinamide, attenuating both ligand-induced and constitutive CCR1-mediated chemotactic recruitment of classical monocytes, disrupting the pro-inflammatory positive feedback loop, thereby exerting therapeutic effects on pSS. These computational predictions provide specific, testable hypotheses for future experimental validation through receptor pharmacology assays and cell-based functional studies.

It should be noted that P. odoratum is a multi-component botanical medicine, and its therapeutic potential in pSS is unlikely to depend on a single active component alone. Beyond Moupinamide, which was identified as the lead compound directly targeting CCR1, our drug-likeness screening revealed additional active components whose predicted targets are functionally associated with the CCL5–CCR1 signaling axis (**Supplementary Table 9**). For example, Coumaroyltyramine (a hydroxycinnamic acid amide structurally related to Moupinamide) is predicted to target JAK2, JAK3, MAPK14, and PIK3CA—key kinases in the JAK-STAT and MAPK pathways that were identified as significantly enriched in our GSEA and KEGG analyses (**Figure 2I**)—suggesting it may attenuate CCR1-mediated intracellular signal transduction through modulation of downstream signaling cascades. Additionally, 4’,5,7-trihydroxy-6,8-dimethyl-homoisoflavanone, a homoisoflavanone with predicted targets including TNF, PTGS1, PTGS2, and ALOX5, may reduce the overall pro-inflammatory milieu driving CCL5 secretion by T/NK cells, thereby attenuating the upstream chemotactic signal. Other components, including steroidal saponin aglycones (e.g., Polygosides a and b) that are predicted to directly target CCR1 along with multiple MAPK and PI3K pathway members, may further contribute through multi-target modulation of the CCL5–CCR1 signaling axis (detailed compound information is provided in **Supplementary Table 9**). This multi-component, multi-target characteristic is consistent with the holistic therapeutic philosophy of traditional Chinese medicine and suggests that P. odoratum may exert synergistic immunomodulatory effects through simultaneously targeting the CCR1 receptor, its downstream signaling cascades, and the upstream inflammatory environment. However, the specific synergistic interactions among these components require systematic validation through combination pharmacology experiments and network-level dose–response studies in future research.

From a clinical translation perspective, several lines of evidence support the therapeutic potential of P. odoratum in pSS-related conditions. First, P. odoratum has a long history of clinical use in traditional Chinese medicine for treating “dryness syndrome” (Zao Bi), the TCM diagnostic category encompassing pSS, and is included in multiple classical formulations for nourishing yin, promoting fluid production, and moistening dryness (39). Published clinical studies have reported that TCM formulations containing yin-nourishing herbs (including P. odoratum) improved subjective symptoms of xerostomia and xerophthalmia in pSS patients, with concurrent reductions in inflammatory markers such as ESR, CRP, and IgG (40). Second, modern pharmacological studies have demonstrated that P. odoratum extracts and their bioactive components—including polysaccharides, steroidal saponins, and phenylpropanoids—possess significant anti-inflammatory and immunomodulatory activities in both *in vitro* and *in vivo* models, including suppression of pro-inflammatory cytokine release (TNF-α, IL-6, IL-1β), inhibition of NF-κB signaling, and modulation of macrophage polarization (12). Third, P. odoratum polysaccharides have been shown to attenuate inflammatory injury and modulate immune responses through regulation of gut microbiota and suppression of M1 macrophage polarization in animal models (41), which is relevant to the systemic immune dysregulation characteristic of pSS. Nevertheless, we must candidly acknowledge several important limitations regarding the P. odoratum–CCR1 connection: (i) the presence and quantitative abundance of Moupinamide in actual P. odoratum preparations used clinically have not been chemically verified in this study; (ii) no extract standardization of P. odoratum was performed; (iii) neither Moupinamide nor P. odoratum extract was tested in any functional immune assay in this work; and (iv) no randomized controlled clinical trial has yet been conducted to specifically evaluate the efficacy and safety of P. odoratum (either as a single herb or as a standardized extract) in pSS patients. The present study provides molecular-level mechanistic rationale (Moupinamide–CCR1 interaction) that may serve as the scientific foundation for designing such clinical trials in the future.

Despite the multi-dimensional computational characterization of CCR1’s candidate role in pSS immunopathology, this study has the following limitations that should be considered when interpreting conclusions: first, all analyses were based on peripheral blood samples, unable to directly reflect local pathology of target organs such as salivary and lacrimal glands; second, in Milo differential abundance analysis, although 59.1% of neighborhoods showed pSS enrichment trends, none reached the strict statistical significance threshold (minimum SpatialFDR = 0.107), likely affected by sample size limitations; third, SCENIC correlation coefficients between regulons and CCR1 were modest (R ≈ 0.16–0.20), requiring experimental validation; fourth, molecular docking and virtual knockout are computational predictions requiring *in vitro* and *in vivo* experimental confirmation; fifth, the cross-sectional design can only establish correlations rather than causation; sixth, P. odoratum has complex components, and additional targets beyond CCR1 may exist; seventh, the RT-qPCR validation cohort was assembled from a clinical sample collection compiled several years ago, and detailed treatment exposure (including corticosteroid and immunosuppressant use), disease duration, infection status, overlap autoimmunity, and standardized glandular/extraglandular activity measures were not systematically recorded at the time of collection. We therefore cannot retrospectively report these variables reliably. We acknowledge that medication and disease-stage effects on chemokine-receptor expression have been reported in autoimmune disease (42, 43) and cannot be excluded as confounders. This cohort serves the narrow purpose of corroborating the direction of CCR1 transcript change observed in the independent public datasets; the principal inferences derive from the public transcriptomic and single-cell data. Future research will focus on: (1) validating CCR1 expression in pSS salivary gland biopsy tissue to determine whether peripheral blood findings extend to the target organ level; (2) conducting *in vitro* pharmacological characterization of the Moupinamide–CCR1 interaction through radioligand binding assays, GTPγS functional assays, β-arrestin recruitment assays, and transwell chemotaxis inhibition experiments to determine whether Moupinamide acts as a competitive antagonist, inverse agonist, or both, and whether it effectively blocks CCL5-induced monocyte migration; (3) establishing animal models to evaluate the efficacy of CCR1 antagonists or P. odoratum extract/Moupinamide in ameliorating pSS-like disease, with assessment of glandular inflammation, salivary flow, and immune cell infiltration; (4) utilizing single-cell ATAC-seq to further verify STAT1/EGR1 epigenetic regulation of CCR1; (5) systematic evaluation of the synergistic effects of multiple P. odoratum active components through combination pharmacology experiments targeting the CCL5–CCR1 signaling axis; and (6) conducting prospective clinical studies to evaluate the efficacy and safety of P. odoratum-containing formulations in pSS patients, with CCR1 expression as a potential pharmacodynamic biomarker.

In summary, this study, through a progressive research strategy of “discovery–localization–subtyping–differentiation–regulation–communication–intervention,” provides exploratory multi-dimensional computational evidence consistent with a candidate role of CCR1 in pSS immunopathology: CCR1 is specifically highly expressed in classical monocytes of pSS patients, predicted to be associated with STAT1/EGR1 regulatory activity at the transcriptional level, and participates in pro-inflammatory monocyte recruitment through the CCL5–CCR1 chemotactic axis; pseudotime analysis revealed that monocyte differentiation trajectory bias in pSS is closely associated with CCR1 expression dynamics. CCR1 shows preliminary potential as a candidate biomarker (AUC = 0.758, moderate discriminatory capacity) warranting further clinical validation. Moupinamide from P. odoratum shows predicted binding affinity to CCR1, offering a computational hypothesis for the “yin-nourishing and dryness-moistening” therapeutic principle that warrants experimental validation, and generating testable hypotheses for future experimental validation in the context of pSS therapeutics.

## Data Availability

The datasets presented in this study can be found in online repositories. The names of the repository/repositories and accession number(s) can be found in the article/**Supplementary Material**.
