# Supplementary figures and images for: CCR1-mediated monocyte chemotaxis in the immunopathology of primary Sjögren’s syndrome: multi-omics integration analysis and computational target prioritization implicating Polygonatum odoratum
Source: Front Immunol. 2026 Jul 20;17:1867098. doi: 10.3389/fimmu.2026.1867098 (PMC13429757; doi:10.3389/fimmu.2026.1867098)

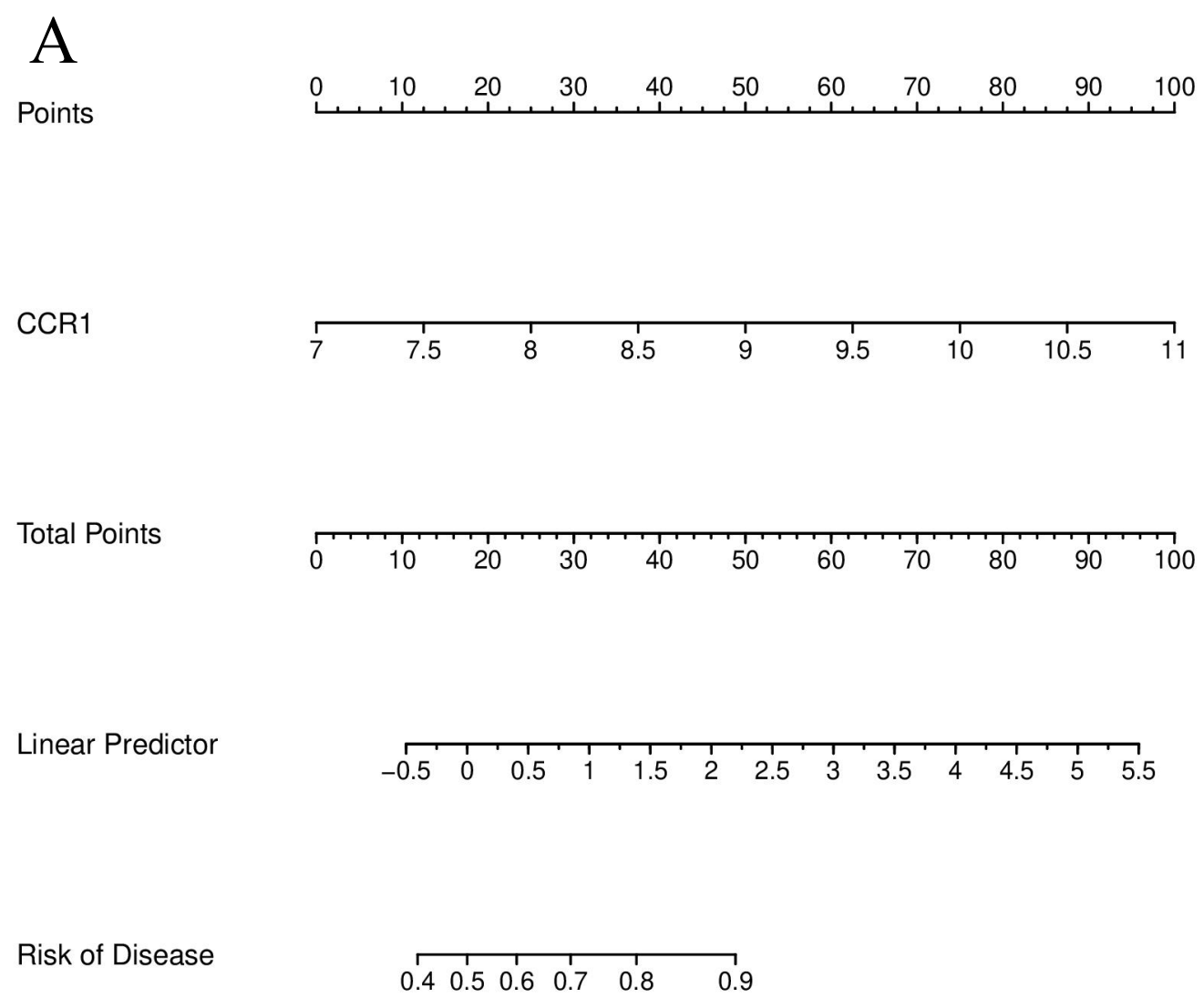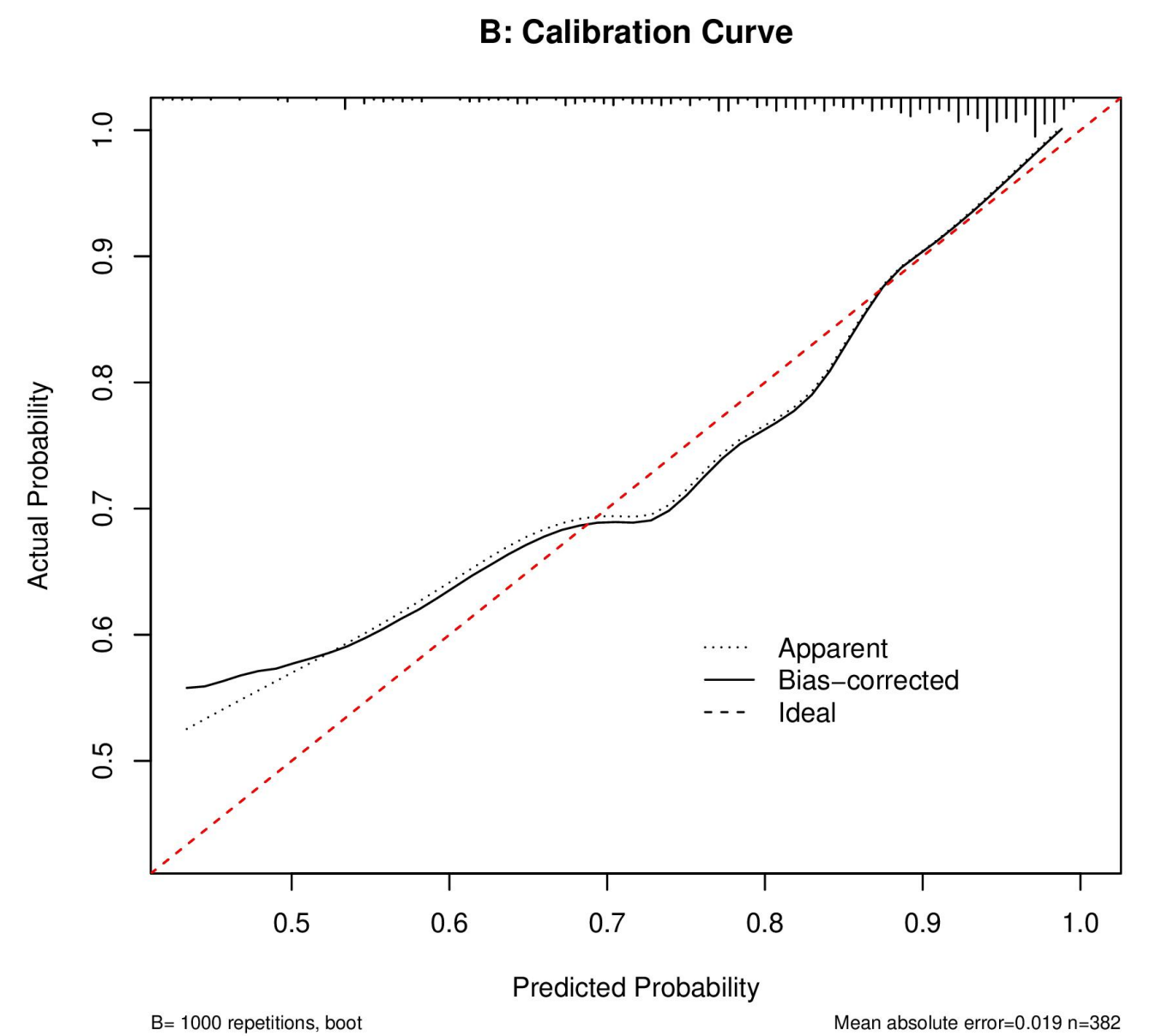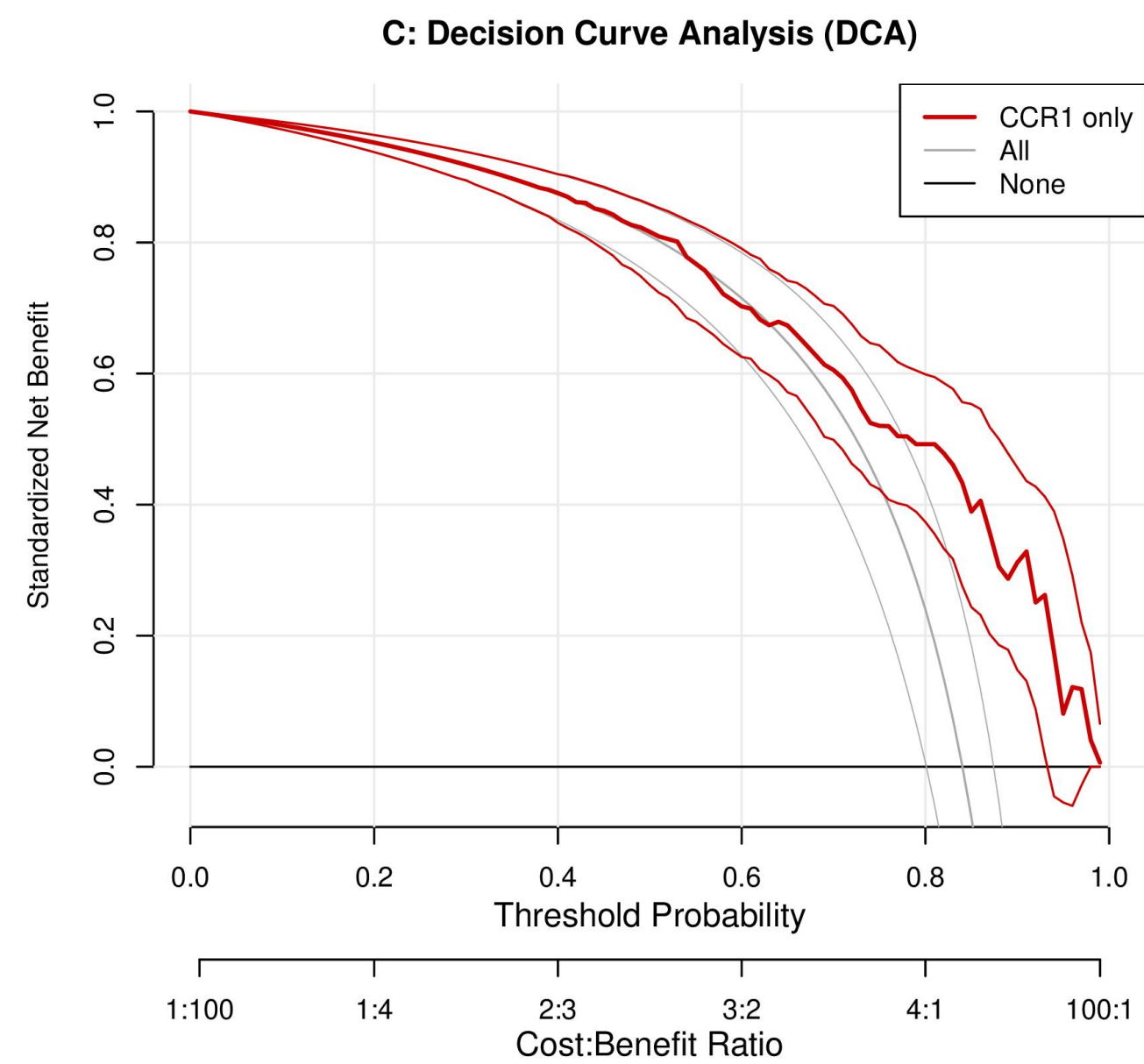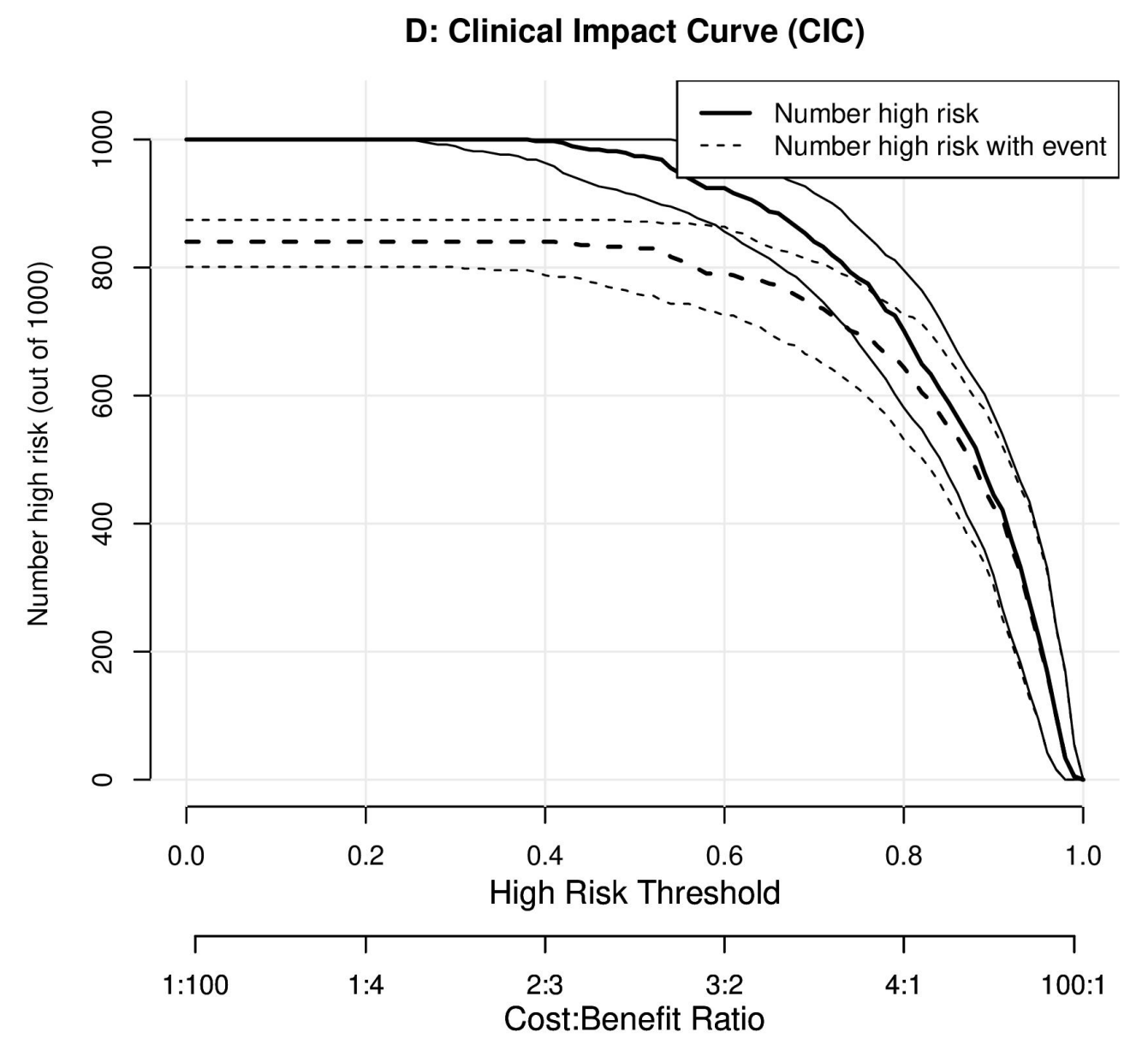

Supplement: Supplementary file 1 [file DataSheet1.pdf]

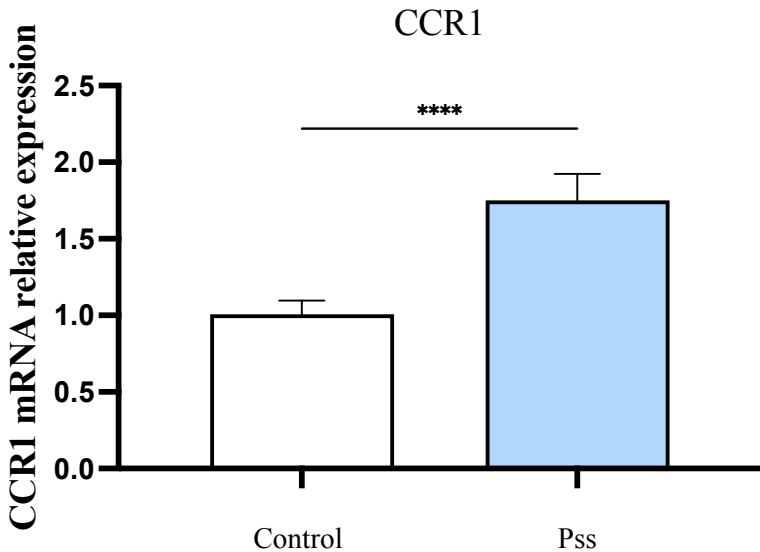

Supplement: Supplementary file 2 [file DataSheet2.pdf]

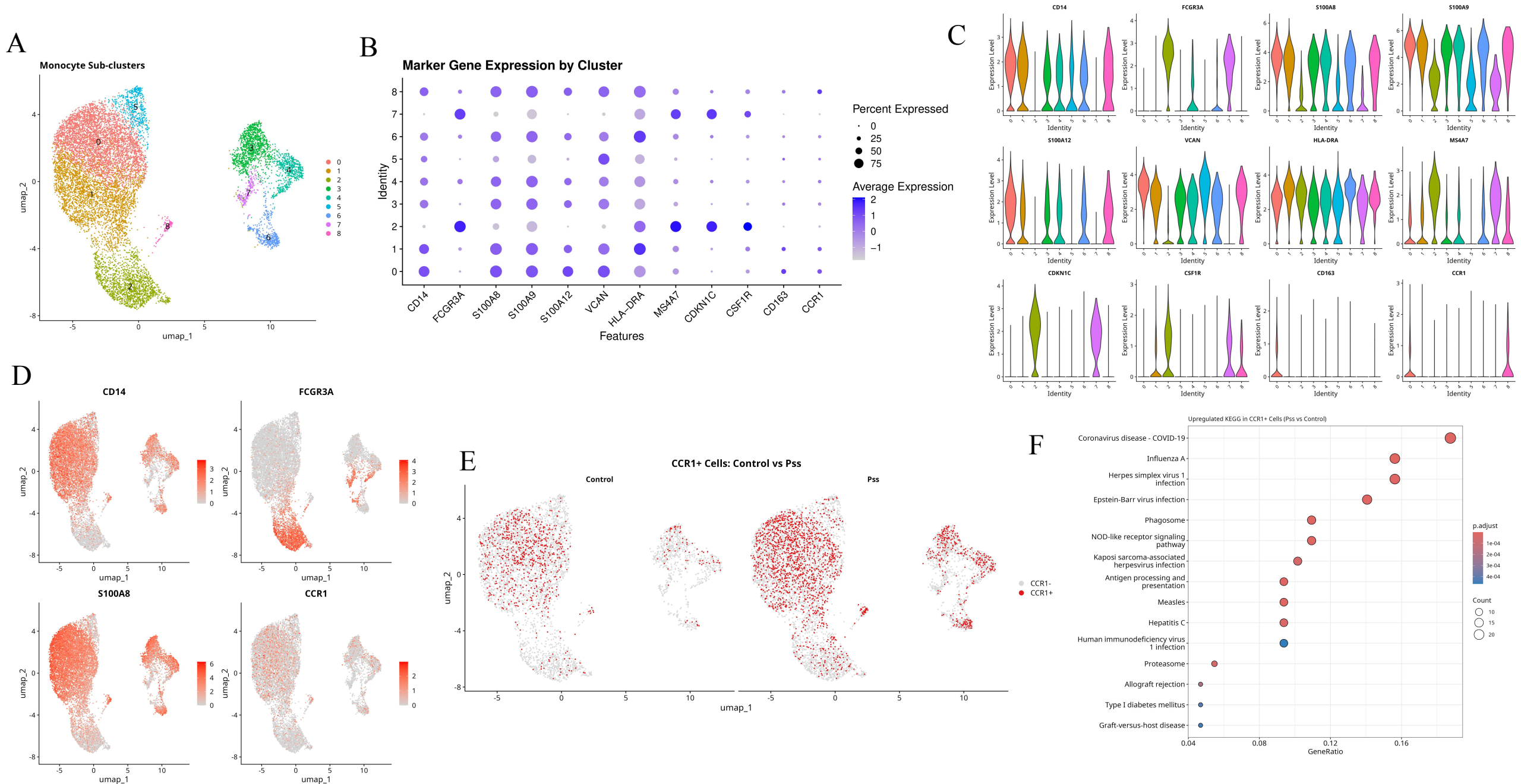

Supplement: Supplementary file 3 [file DataSheet3.pdf]

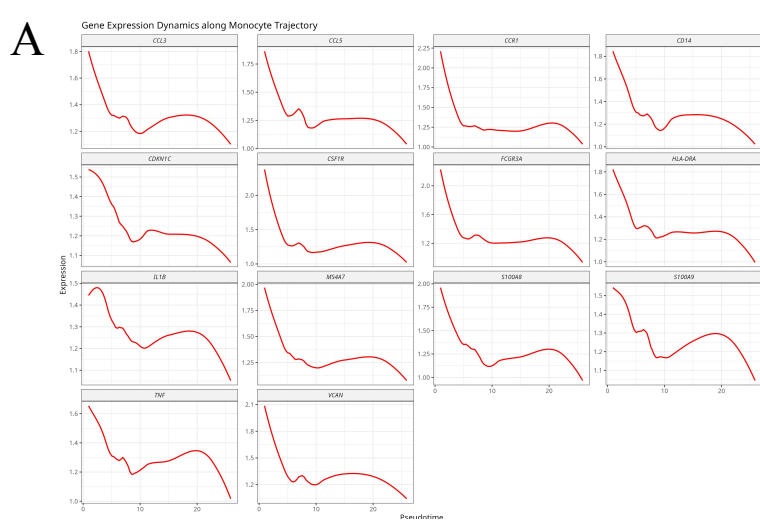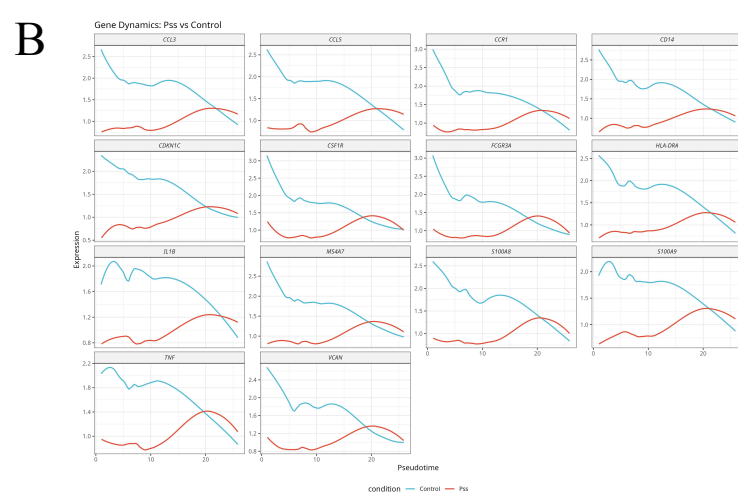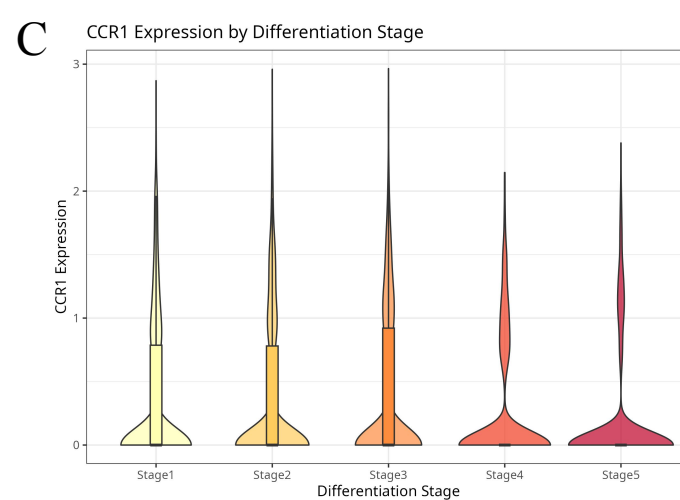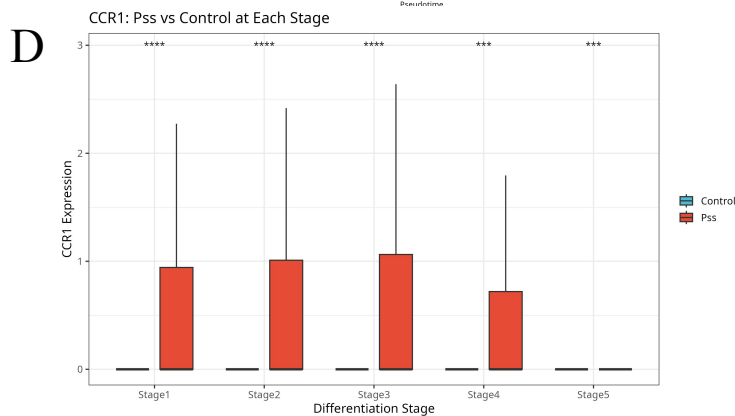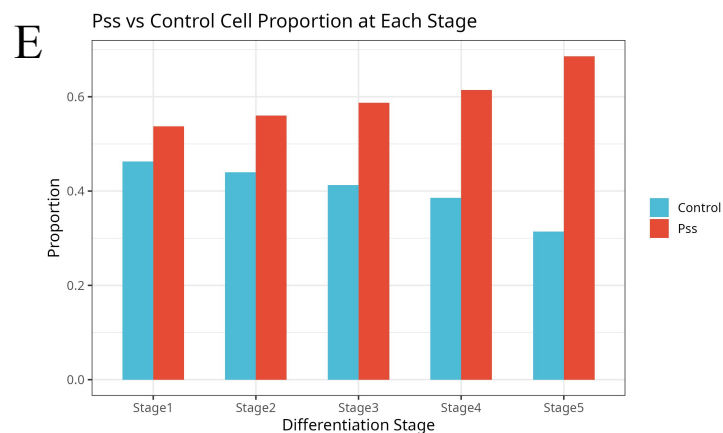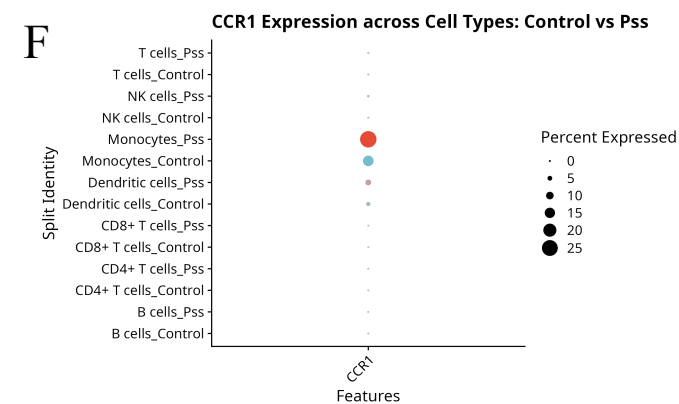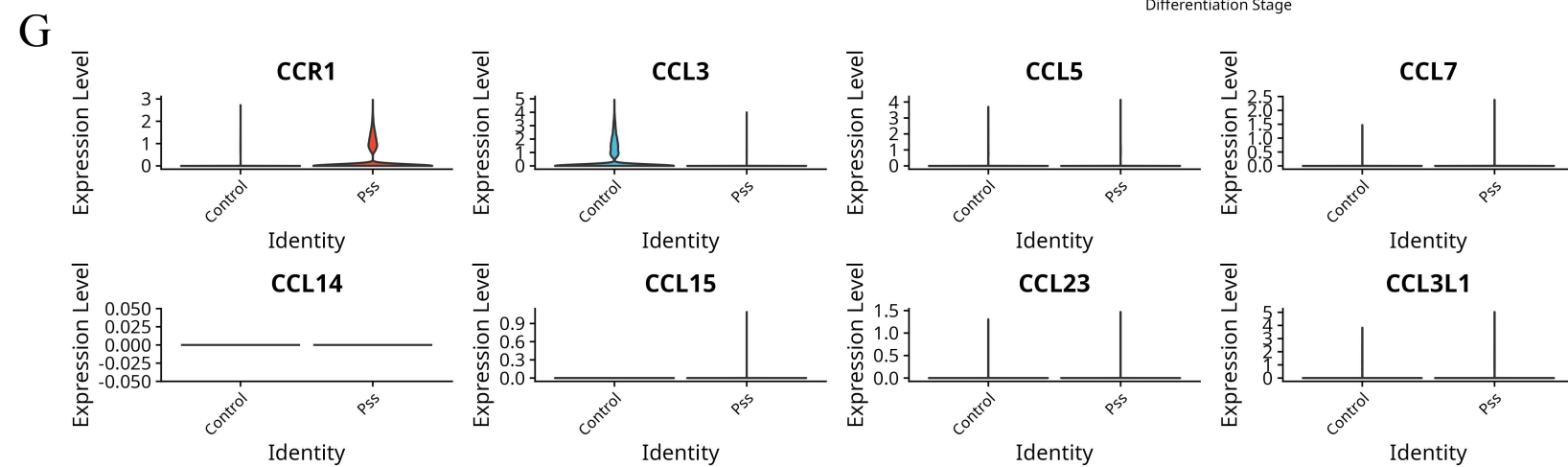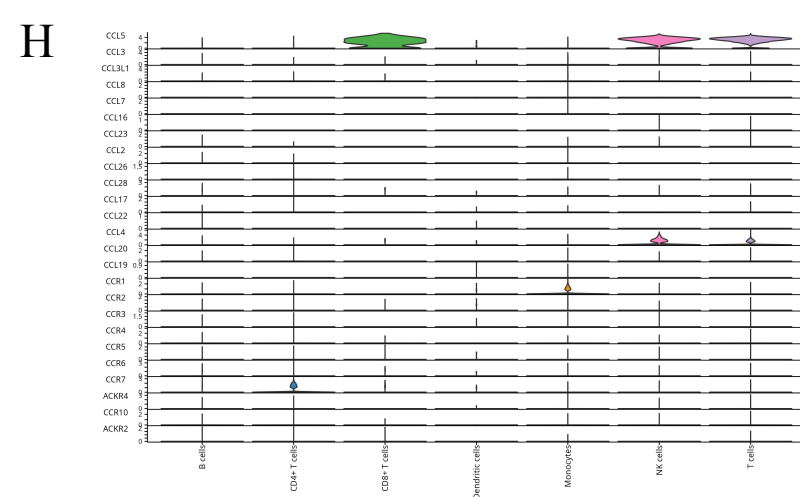

Supplement: Supplementary file 4 [file DataSheet4.pdf]
