# Supplementary material for: CCR1-mediated monocyte chemotaxis in the immunopathology of primary Sjögren’s syndrome: multi-omics integration analysis and computational target prioritization implicating Polygonatum odoratum
Source: Front Immunol. 2026 Jul 20;17:1867098. doi: 10.3389/fimmu.2026.1867098 (PMC13429757; doi:10.3389/fimmu.2026.1867098)
Supplement: Supplementary file 6 [file DataSheet6.docx]

# Supplementary Figure Legends

## Supplementary Figure 1. Nomogram Diagnostic Model Construction and Performance Evaluation Based on CCR1 Expression

**Supplementary Figure 1A.** Nomogram for pSS risk prediction based on CCR1 expression level. The upper scale represents variable score (Points), CCR1 expression values are mapped to corresponding scores, and the total score is converted through the Linear Predictor to yield individual pSS disease risk probability (Risk of Disease).

**Supplementary Figure 1B.** Calibration curve of the nomogram model. The x-axis represents predicted probability, the y-axis represents actual probability. The dashed gray line represents ideal model prediction (Ideal), and the solid line represents bias-corrected model performance (Bias-corrected). Mean absolute error MAE = 0.019 (n = 382, B = 1000 Bootstrap resampling), indicating excellent prediction accuracy.

**Supplementary Figure 1C.** Decision curve analysis (DCA). The red curve represents the CCR1-only model, the gray curve represents the treat-all strategy (All), and the black line represents the treat-none strategy (None). Results show that the CCR1 model provides significant standardized net benefit across a wide range of threshold probabilities.

**Supplementary Figure 1D.** Clinical impact curve (CIC). The solid line (Number high risk) represents the number of individuals classified as high risk by the model, and the dashed line (Number high risk with event) represents the number of actual pSS patients among them, demonstrating clinical discrimination performance across different high risk thresholds.

## Supplementary Figure 2. RT-qPCR Validation of CCR1 Expression in Clinical Samples

**Supplementary Figure 2.** RT-qPCR validation of CCR1 relative expression in PBMCs from pSS patients (n = 65) and healthy controls (n = 48). The bar chart shows CCR1 mRNA relative expression levels in the Control and Pss groups detected by RT-qPCR. GAPDH served as the internal reference. Data are presented as Mean ± SD. **** indicates P < 0.0001. Results are consistent with bioinformatics predictions, confirming significant CCR1 overexpression in PBMCs of pSS patients at the clinical sample level.

## Supplementary Figure 3. Monocyte Sub-clustering Details and CCR1-Positive Cell Analysis

**Supplementary Figure 3A.** UMAP plot of monocyte sub-clusters (Monocyte Sub-clusters). A total of 9 sub-clusters (numbered 0–8) were identified after re-clustering, each marked with a distinct color.

**Supplementary Figure 3B.** Bubble plot of marker gene expression by cluster (Marker Gene Expression by Cluster). Marker genes include CD14, FCGR3A, S100A8, S100A9, S100A12, VCAN, HLA-DRA, MS4A7, CDKN1C, CSF1R, CD163, and CCR1. Bubble size represents the percentage of cells expressing each gene (Percent Expressed), and color intensity represents average expression level (Average Expression).

**Supplementary Figure 3C.** Violin plots of marker gene expression by cluster. Shows the expression distribution of CD14, FCGR3A, S100A8, S100A9, S100A12, VCAN, HLA-DRA, MS4A7, CDKN1C, CSF1R, CD163, and CCR1 across each sub-cluster.

**Supplementary Figure 3D.** FeaturePlots of key marker genes (CD14, FCGR3A, S100A8, CCR1) on the monocyte UMAP. Deeper red indicates higher expression. CD14 is broadly highly expressed in classical clusters, FCGR3A is specifically highly expressed in non-classical clusters, and CCR1 is primarily concentrated in a subset of classical clusters.

**Supplementary Figure 3E.** Split UMAP distribution of CCR1-positive/negative cells in pSS and Control groups (CCR1+ Cells: Control vs Pss). Red represents CCR1+ cells (expression > 0), gray represents CCR1− cells. CCR1+ cells show denser distribution in classical monocyte regions in the pSS group compared to the Control group.

**Supplementary Figure 3F.** KEGG pathway enrichment bubble plot of upregulated genes in CCR1-positive monocytes (Upregulated KEGG in CCR1+ Cells, Pss vs Control). Enriched pathways include Coronavirus disease - COVID-19, Influenza A, Herpes simplex virus 1 infection, Epstein-Barr virus infection, Phagosome, NOD-like receptor signaling pathway, and Antigen processing and presentation. Bubble size represents gene count (Count), and color represents adjusted P value (p.adjust).

## Supplementary Figure 4. Pseudotime Supplementary Analysis and CCR1 Ligand Expression Profiles

**Supplementary Figure 4A.** Multi-gene expression dynamics along the monocyte pseudotime trajectory (Gene Expression Dynamics along Monocyte Trajectory). Shows expression trends of CCR1, CCR2, CD14, CD1E, CX3CR1, CD1B, FCGR3A, NLRP3A, CSF1, ADAM17, S100A8, and S100A9 along pseudotime.

**Supplementary Figure 4B.** Multi-gene pseudotime dynamics comparing pSS vs Control (Gene Dynamics: Pss vs Control). Red lines represent the pSS group and blue lines represent the Control group, showing differences in pseudotime expression dynamics between groups.

**Supplementary Figure 4C.** CCR1 expression by differentiation stage (Differentiation Stage 1–5) violin plot (CCR1 Expression by Differentiation Stage). CCR1 shows clear expression in Stage 1–3, approaching silencing in Stage 4–5.

**Supplementary Figure 4D.** CCR1 expression comparison between pSS and Control at each differentiation stage (CCR1: Pss vs Control at Each Stage). CCR1 in the pSS group is significantly higher than Control at Stage 1–2 (****, P < 0.0001), Stage 3 (***, P < 0.001), and Stage 4 (***, P < 0.001), further confirming disease-specific CCR1 upregulation during early-to-mid differentiation.

**Supplementary Figure 4E.** Cell proportion bar chart of each differentiation stage in pSS vs Control groups (Pss vs Control Cell Proportion at Each Stage). The pSS group (red) shows increased proportions in Stage 3–5 compared to the Control group (blue), suggesting a shift toward mid-to-late differentiation stages in pSS.

**Supplementary Figure 4F.** Split bubble plot of CCR1 expression across all immune cell types comparing Control vs Pss (CCR1 Expression across Cell Types: Control vs Pss). Displays CCR1 expression percentage and average expression by cell type (B cells, CD4+ T cells, CD8+ T cells, dendritic cells, monocytes, NK cells, T cells) and group, reconfirming monocytes as the primary CCR1-expressing cells with higher expression in pSS.

**Supplementary Figure 4G.** Violin plots of CCR1 and its ligands (CCL3, CCL5, CCL7, CCL14, CCL15, CCL23, CCL3L1) in monocytes comparing pSS vs Control. CCR1 is significantly upregulated in pSS monocytes, and CCL3 also shows some degree of upregulation.

**Supplementary Figure 4H.** Bubble plot of all CCL chemokine family members (CCL ligands and CCR receptors) across immune cell types (B cells, CD4+ T cells, CD8+ T cells, dendritic cells, monocytes, NK cells, T cells). Shows CCL5 primarily highly expressed in T cells and NK cells, while CCR1 is specifically highly expressed in monocytes.

## Supplementary Figure 5. SCENIC Transcription Factor Supplementary Analysis and CellChat Detailed Communication Analysis

**Supplementary Figure 5A.** Regulon activity clustered heatmap by monocyte subtype (Regulon Activity by Monocyte Subtype: Classical CD14++CD16−, Non-classical CD14+CD16++, Intermediate CD14++CD16+). Left-side red marks indicate CCR1_upstream transcription factors. The heatmap reveals differences in transcription factor regulatory networks among functional subtypes.

**Supplementary Figure 5B.** UMAP distribution of top key transcription factor (EGR1_extended, STAT2, STAT1, STAT2_extended, STAT1_extended, CEBPD_extended) regulon activity on the monocyte UMAP. Deeper red indicates higher AUCell scores.

**Supplementary Figure 5C.** Violin plots comparing regulon activity of top key transcription factors between pSS and Control groups. Shows activity differences of EGR1_extended, STAT2_extended, STAT1, STAT2, STAT1_extended, and CEBPD_extended between groups, with all transcription factors showing significantly higher activity in the pSS group.

**Supplementary Figure 5D.** Overall comparison bar charts of interaction number and strength between pSS and Control groups (Number of Interactions and Interaction Strength). The pSS group shows higher interaction numbers (Control: 155 vs Pss: 196) and strength (Control: 3.48 vs Pss: 4.215) compared to Control.

**Supplementary Figure 5E.** Bubble plot of ligand-receptor signals directed toward monocytes (Signal to Monocytes: Control vs Pss). Shows changes in signaling pathways targeting monocytes between groups, with MIF-(CD74+CD44) and MIF-(CD74+CXCR4) pathways active in pSS.

**Supplementary Figure 5F.** Bubble plot of signals from monocytes (Signals from Monocytes: Control vs Pss). Shows changes in signaling pathways originating from monocytes between groups.

**Supplementary Figure 5G.** Signaling pathway relative information flow ranking comparison (Signaling Pathway Ranking: Pss vs Control). Red/pink represents pSS, blue/cyan represents Control. Shows relative strength changes of MIF, GALECTIN, CypA, ANNEXIN, TGFb, CCL, BAFF, ANGPTL, BTLA, and other pathways between groups.

**Supplementary Figure 5H.** MIF signaling pathway network circle plot in the pSS group (MIF Signaling – Pss). Line thickness represents MIF signal communication strength between cell types. Dendritic cells serve as the core hub, with extensive MIF signal interactions with B cells, T cells, and monocytes.

**Supplementary Figure 5I.** Outgoing signaling patterns heatmap in the pSS group. Shows the relative contribution of each cell type (B cells, CD4+ T cells, CD8+ T cells, dendritic cells, monocytes, NK cells, T cells) as signal senders across MIF, GALECTIN, CypA, ANNEXIN, TGFb, CCL, BAFF, ANGPTL, FLT3, and BTLA pathways.

**Supplementary Figure 5J.** Incoming signaling patterns heatmap in the pSS group. Shows the relative contribution of each cell type as signal receivers across the above pathways. B cells and monocytes are the main receivers of MIF and BAFF signals, while CCL pathway signals are primarily received by monocytes and dendritic cells.
