# Supplementary material for: CCR1-mediated monocyte chemotaxis in the immunopathology of primary Sjögren’s syndrome: multi-omics integration analysis and computational target prioritization implicating Polygonatum odoratum
Source: Front Immunol. 2026 Jul 20;17:1867098. doi: 10.3389/fimmu.2026.1867098 (PMC13429757; doi:10.3389/fimmu.2026.1867098)
Supplement: Supplementary file 7 [file DataSheet7.docx]

# Supplementary Table Legends

## Supplementary Table 1. Marker Genes for Each Monocyte Sub-cluster

Supplementary Table 1 lists the marker genes for each monocyte sub-cluster (clusters 0–8) identified after sub-clustering analysis. Positive marker genes satisfying min.pct = 0.25 and log2FC > 0.25 were retained using the FindAllMarkers function. The table contains the following fields: differential expression P value (p_val), average log2 fold change (avg_log2FC), percentage of cells expressing the gene within the cluster (pct.1), percentage of cells expressing the gene in other clusters (pct.2), adjusted P value (p_val_adj), cluster number (cluster), and gene name (gene). A total of 6,309 marker genes were identified, providing the basis for subtype annotation.

## Supplementary Table 2. Milo Differential Abundance Analysis Results

Supplementary Table 2 lists the results of Milo differential abundance analysis of monocytes. Milo detects abundance differences in cell neighborhoods between pSS and Control groups without pre-defined cluster assignments. The table contains: log fold change (logFC), log average counts per million (logCPM), F statistic (F), raw P value (PValue), FDR-corrected P value (FDR), neighborhood number (Nhood), spatially corrected FDR (SpatialFDR), predominant monocyte subtype of the neighborhood (mono_subtype), and the fraction of that subtype (mono_subtype_fraction). A total of 996 neighborhoods were detected, of which 589 (59.1%) showed positive logFC values (higher abundance in pSS).

## Supplementary Table 3. Complete List of Differentially Expressed Genes in Monocytes: pSS vs Control

Supplementary Table 3 lists the complete differentially expressed genes between pSS patients and healthy controls in monocytes. A total of 280 significant DEGs were identified, including 183 upregulated and 97 downregulated genes. The table contains: differential expression P value (p_val), average log2 fold change (avg_log2FC), percentage of cells expressing in pSS group (pct.1), percentage in Control group (pct.2), adjusted P value (p_val_adj), gene name (gene), and differential direction (sig: Up/Down). Top upregulated genes include IFI44L (log2FC = 3.17), IFI6 (log2FC = 2.61), and ISG15 (log2FC = 2.61). CCR1 was also among the significantly upregulated genes (log2FC = 1.15, p_val_adj = 2.79 × 10⁻⁴⁸).

## Supplementary Table 4. Differentially Expressed Genes in CCR1-Positive Monocytes

Supplementary Table 4 lists differentially expressed genes between CCR1-positive (CCR1+) and CCR1-negative (CCR1−) monocytes in the pSS group. A total of 4,701 genes were analyzed, with 219 significantly upregulated and 330 significantly downregulated. The table contains: P value (p_val), average log2 fold change (avg_log2FC), percentage of CCR1+ cells expressing (pct.1), percentage of CCR1− cells expressing (pct.2), adjusted P value (p_val_adj), and gene name (gene). Top upregulated genes are dominated by interferon response genes including IFI44L (log2FC = 3.33), IFI6 (log2FC = 2.67), and LY6E (log2FC = 2.30).

## Supplementary Table 5. Upstream Transcription Factors Directly Regulating CCR1

Supplementary Table 5 lists the upstream transcription factors identified by SCENIC analysis as directly targeting the CCR1 gene promoter region. The table contains two fields: target gene name (target_gene) and upstream transcription factor name (upstream_TF). Results show that CCR1 is directly regulated by four regulons: STAT1, EGR1_extended, FOS_extended, and STAT1_extended. Notably, STAT1 appears in both STAT1 and STAT1_extended regulon forms, suggesting highly robust regulation of CCR1.

## Supplementary Table 6. Correlation Between Regulons and CCR1 Expression

Supplementary Table 6 lists the Spearman correlation analysis results between all regulon activity scores and CCR1 expression levels. The table contains: regulon name with target gene count (regulon), Spearman correlation coefficient (correlation), P value (pvalue), and adjusted P value (padj). Results are ranked by correlation coefficient in descending order. The top five are: EGR1_extended (41 target genes, R = 0.204), STAT2 (36 target genes, R = 0.199), STAT1 (117 target genes, R = 0.198), STAT2_extended (42 target genes, R = 0.198), and STAT1_extended (136 target genes, R = 0.196), all showing highly significant positive correlations (padj ≈ 0).

## Supplementary Table 7. Regulon Activity Differences Between pSS and Control Groups

Supplementary Table 7 lists the regulon activity differential analysis results between pSS and Control groups. The table contains: regulon name (regulon), mean AUCell score in pSS (mean_Pss), mean AUCell score in Control (mean_Control), P value (pvalue), log fold change (logFC), and adjusted P value (padj). Results are ranked by logFC in descending order. The most significantly upregulated regulons in pSS are: IRF7_extended (59 target genes, logFC = 2.19), IRF7 (50 target genes, logFC = 2.34), STAT2_extended (42 target genes, logFC = 2.03), and STAT2 (36 target genes, logFC = 2.04), all reaching extreme significance (padj ≈ 0).

## Supplementary Table 8. Key Transcription Factors Positively Correlated with CCR1 and Significantly Activated in pSS

Supplementary Table 8 lists key transcription factors simultaneously meeting two criteria: (1) regulon activity positively correlated with CCR1 expression; (2) regulon activity significantly upregulated in the pSS group. The table contains: regulon name (regulon), correlation coefficient with CCR1 (correlation), correlation P value and padj (pvalue.cor, padj.cor), mean activity in pSS (mean_Pss), mean activity in Control (mean_Control), differential P value and padj (pvalue.diff, padj.diff), and logFC. A total of 10 synergistic transcription factors were identified. EGR1_extended (R = 0.204, logFC = 0.48) ranked first in CCR1 correlation; STAT2 (R = 0.199, logFC = 2.04) and STAT1 (R = 0.198, logFC = 1.48) combined high correlation with high disease differential; IRF7 (R = 0.165, logFC = 2.34) showed the highest activity upregulation in pSS. These transcription factors collectively constitute the upstream regulatory network driving CCR1 aberrant upregulation.

## Supplementary Table 9. Active Components of P. odoratum Passing Drug-Likeness Screening and Their Predicted Relevance to the CCL5–CCR1 Signaling Axis

Supplementary Table 9 lists all active components of Polygonatum odoratum that passed the triple drug-likeness screening criteria (SwissADME: high gastrointestinal absorption, Lipinski's Rule of Five with no more than one violation, and Veber's rules compliance). A total of 24 compounds were retained. The table contains the following fields: compound name, HERB 2.0 ingredient ID (Ingredient ID), molecular formula (Molecular Formula), molecular weight in Daltons (MW), consensus LogP (Consensus LogP), number of hydrogen bond donors (HBD), number of hydrogen bond acceptors (HBA), topological polar surface area in Å² (TPSA), number of rotatable bonds (Rotatable Bonds), number of Lipinski rule violations (Lipinski Violations), gastrointestinal absorption classification (GI Absorption), number of predicted targets from SwissTargetPrediction (Predicted Targets), and the relevance of predicted targets to the CCL5–CCR1 signaling axis (CCL5–CCR1 Axis Relevance). Target relevance was classified as: "Direct" if the compound's predicted targets include CCR1 itself; "Downstream signaling" if targets include kinases or transcription factors in CCR1 downstream pathways (JAK-STAT, MAPK, PI3K-AKT); "Anti-inflammatory" if targets include inflammation-related molecules (COX, LOX, TNF, NF-κB pathway); or "No direct pathway relevance identified." Moupinamide, identified as the lead compound directly targeting CCR1 by molecular docking (binding energy = −8.1 kcal/mol), is highlighted. Additionally, three steroidal saponin compounds (25(R)-Spirost-5-en-3β-ol aglycone, Polygosides A aglycone, Polygosides B aglycone) were also predicted to directly target CCR1, suggesting potential multi-component synergistic effects on the CCL5–CCR1 axis.
