# Supplementary material for: CCR1-mediated monocyte chemotaxis in the immunopathology of primary Sjögren’s syndrome: multi-omics integration analysis and computational target prioritization implicating Polygonatum odoratum
Source: Front Immunol. 2026 Jul 20;17:1867098. doi: 10.3389/fimmu.2026.1867098 (PMC13429757; doi:10.3389/fimmu.2026.1867098)
Supplement: Supplementary file 8 [file DataSheet8.docx]

################################################################################

#

# Supplementary Code

#

# CCR1-Mediated Monocyte Chemotaxis in the Immunopathology of Primary

# Sjögren's Syndrome: Multi-Omics Integration Analysis and Computational

# Target Prioritization Implicating Polygonatum odoratum

##

# Datasets used:

# Training: GSE51092, GSE66795

# Validation: GSE84844

# Single-cell: GSE253568

#

################################################################################

# ==============================================================================

# SECTION 1: Batch Effect Correction (ComBat)

# Input: Individual dataset expression matrices (.txt)

# Output: merge.preNorm.txt, merge.normalize.txt

# ==============================================================================

library(limma)

library(sva)

# Get all .txt files in the directory

files <- dir()

files <- grep("txt$", files, value = TRUE)

geneList <- list()

# Load gene information from each file

for (file in files) {

if (file %in% c("merge.preNorm.txt", "merge.normalize.txt")) next

rt <- read.table(file, header = TRUE, sep = "\t", check.names = FALSE)

geneNames <- as.vector(rt[, 1])

uniqGene <- unique(geneNames)

header <- unlist(strsplit(file, "\\.|\\-"))

geneList[[header[1]]] <- uniqGene

}

# Identify common genes across all datasets

interGenes <- Reduce(intersect, geneList)

# Combine data from all files based on common genes

allTab <- data.frame()

batchType <- c()

for (i in 1:length(files)) {

inputFile <- files[i]

if (inputFile %in% c("merge.preNorm.txt", "merge.normalize.txt")) next

header <- unlist(strsplit(inputFile, "\\.|\\-"))

rt <- read.table(inputFile, header = TRUE, sep = "\t", check.names = FALSE)

rt <- as.matrix(rt)

rownames(rt) <- rt[, 1]

exp <- rt[, 2:ncol(rt)]

dimnames <- list(rownames(exp), colnames(exp))

data <- matrix(as.numeric(as.matrix(exp)), nrow = nrow(exp), dimnames = dimnames)

rt <- avereps(data)

colnames(rt) <- paste0(header[1], "_", colnames(rt))

if (i == 1) {

allTab <- rt[interGenes, ]

} else {

allTab <- cbind(allTab, rt[interGenes, ])

}

batchType <- c(batchType, rep(i, ncol(rt)))

}

# Save combined data before normalization

outTab <- rbind(geneNames = colnames(allTab), allTab)

write.table(outTab, file = "merge.preNorm.txt", sep = "\t", quote = FALSE, col.names = FALSE)

# Apply ComBat normalization to adjust batch effects

outTab <- ComBat(allTab, batchType, par.prior = TRUE)

outTab <- rbind(geneNames = colnames(outTab), outTab)

write.table(outTab, file = "merge.normalize.txt", sep = "\t", quote = FALSE, col.names = FALSE)

# ==============================================================================

# SECTION 2: PCA Visualization (Before and After Batch Correction)

# ==============================================================================

library(ggplot2)

library(ggpubr)

bioPCA <- function(inputFile = NULL, outFile = NULL, titleName = NULL) {

rt <- read.table(inputFile, header = TRUE, sep = "\t", check.names = FALSE, row.names = 1)

data <- t(rt)

Project <- gsub("(.*?)\\_.*", "\\1", rownames(data))

data.pca <- prcomp(data)

pcaPredict <- predict(data.pca)

PCA <- data.frame(PC1 = pcaPredict[, 1], PC2 = pcaPredict[, 2], Type = Project)

pdf(file = outFile, width = 5.5, height = 4.25)

p1 <- ggscatter(data = PCA, x = "PC1", y = "PC2", color = "Type", shape = "Type",

ellipse = TRUE, ellipse.type = "norm", ellipse.border.remove = FALSE,

ellipse.alpha = 0.1, size = 2, main = titleName, legend = "right") +

theme(plot.margin = unit(rep(1.5, 4), 'lines'), plot.title = element_text(hjust = 0.5))

print(p1)

dev.off()

}

bioPCA(inputFile = "merge.preNorm.txt", outFile = "PCA.preNorm.pdf", titleName = "Before batch correction")

bioPCA(inputFile = "merge.normalize.txt", outFile = "PCA.normalize.pdf", titleName = "After batch correction")

# ==============================================================================

# SECTION 3: Differential Expression Analysis + Heatmap + Volcano Plot

# ==============================================================================

library(limma)

library(pheatmap)

library(ggplot2)

library(ggrepel)

library(dplyr)

logFCfilter <- 0.585

adj.P.Val.Filter <- 0.05

inputFile <- "merge.normalize.txt"

rt <- read.table(inputFile, header = TRUE, sep = "\t", check.names = FALSE)

rt <- as.matrix(rt)

rownames(rt) <- rt[, 1]

exp <- rt[, 2:ncol(rt)]

dimnames <- list(rownames(exp), colnames(exp))

data <- matrix(as.numeric(as.matrix(exp)), nrow = nrow(exp), dimnames = dimnames)

data <- avereps(data)

Type <- gsub("(.*)\\_(.*)\\_(.*)", "\\3", colnames(data))

data <- data[, order(Type)]

Project <- gsub("(.+)\\_(.+)\\_(.+)", "\\1", colnames(data))

Type <- gsub("(.*)\\_(.*)\\_(.*)", "\\3", colnames(data))

colnames(data) <- gsub("(.+)\\_(.+)\\_(.+)", "\\2", colnames(data))

design <- model.matrix(~0 + factor(Type))

colnames(design) <- c("Control", "Treat")

fit <- lmFit(data, design)

cont.matrix <- makeContrasts(Treat - Control, levels = design)

fit2 <- contrasts.fit(fit, cont.matrix)

fit2 <- eBayes(fit2)

allDiff <- topTable(fit2, adjust = 'fdr', number = Inf)

allDiffOut <- rbind(id = colnames(allDiff), allDiff)

write.table(allDiffOut, file = "all.txt", sep = "\t", quote = FALSE, col.names = FALSE)

diffSig <- allDiff[with(allDiff, (abs(logFC) > logFCfilter & adj.P.Val < adj.P.Val.Filter)), ]

diffSigOut <- rbind(id = colnames(diffSig), diffSig)

write.table(diffSigOut, file = "diff.txt", sep = "\t", quote = FALSE, col.names = FALSE)

diffGeneExp <- data[row.names(diffSig), ]

diffGeneExpOut <- rbind(id = paste0(colnames(diffGeneExp), "_", Type), diffGeneExp)

write.table(diffGeneExpOut, file = "diffGeneExp.txt", sep = "\t", quote = FALSE, col.names = FALSE)

# Heatmap

geneNum <- 50

diffUp <- diffSig[diffSig$logFC > 0, ]

diffDown <- diffSig[diffSig$logFC < 0, ]

geneUp <- row.names(diffUp)

geneDown <- row.names(diffDown)

if (nrow(diffUp) > geneNum) geneUp <- row.names(diffUp)[1:geneNum]

if (nrow(diffDown) > geneNum) geneDown <- row.names(diffDown)[1:geneNum]

hmExp <- data[c(geneUp, geneDown), ]

names(Type) <- colnames(data)

Type <- as.data.frame(Type)

Type <- cbind(Project, Type)

pdf(file = "heatmap.pdf", width = 10, height = 7)

pheatmap(hmExp, annotation_col = Type,

color = colorRampPalette(c("blue2", "white", "red2"))(50),

cluster_cols = FALSE, show_colnames = FALSE, scale = "row",

fontsize = 8, fontsize_row = 5.5, fontsize_col = 8)

dev.off()

# Volcano plot with gene labels

rt <- read.table("all.txt", header = TRUE, sep = "\t", check.names = FALSE)

if ("id" %in% colnames(rt)) rownames(rt) <- rt$id

Sig <- ifelse((rt$adj.P.Val < adj.P.Val.Filter) & (abs(rt$logFC) > logFCfilter),

ifelse(rt$logFC > logFCfilter, "Up", "Down"), "Not")

rt <- mutate(rt, Sig = Sig)

top_label_genes <- c(

rownames(rt[rt$Sig == "Up", ])[1:min(10, sum(rt$Sig == "Up"))],

rownames(rt[rt$Sig == "Down", ])[1:min(10, sum(rt$Sig == "Down"))]

)

rt$label <- ifelse(rownames(rt) %in% top_label_genes, rownames(rt), "")

p <- ggplot(rt, aes(x = logFC, y = -log10(adj.P.Val))) +

geom_point(aes(color = Sig), size = 1.8) +

scale_color_manual(values = c("blue2", "grey", "red2")) +

geom_label_repel(data = subset(rt, label != ""), aes(label = label),

size = 2.2, box.padding = 0.3, point.padding = 0.3,

segment.color = "grey50", max.overlaps = Inf) +

labs(title = "Volcano Plot", x = "log2(Fold Change)", y = "-log10(Adjusted P-value)") +

theme_bw() + theme(plot.title = element_text(size = 14, hjust = 0.5, face = "bold"))

pdf(file = "vol_labeled.pdf", width = 6, height = 5)

print(p)

dev.off()

# ==============================================================================

# SECTION 4: SwissADME Drug-likeness Screening

# ==============================================================================

inputFile <- "swissadme.csv"

data <- read.csv(inputFile, header = TRUE, sep = ",", check.names = FALSE)

outTab <- subset(data,

MW < 500 &

`#H-bond donors` < 5 &

`#H-bond acceptors` < 10 &

`#Rotatable bonds` < 10 &

(`iLOGP` > -2 & `iLOGP` < 5) &

(`XLOGP3` > -2 & `XLOGP3` < 5) &

(`Consensus Log P` > -2 & `Consensus Log P` < 5))

outTab <- subset(outTab, `GI absorption` == "High")

write.csv(outTab, file = "swissadme.filter.csv", row.names = FALSE)

# ==============================================================================

# SECTION 5: Drug Target Prediction (SwissTargetPrediction)

# ==============================================================================

probFilter <- 0.01

files <- list.files(pattern = "*.csv$")

files <- setdiff(files, "swissadme.filter.csv")

admeRT <- read.csv("swissadme.filter.csv", header = TRUE, sep = ",", check.names = FALSE, row.names = 1)

outTab <- data.frame()

for (inputFile in files) {

rt <- read.csv(inputFile, header = TRUE, sep = ",", check.names = FALSE)

rt <- subset(rt, `Probability*` > probFilter)

geneNames <- unlist(strsplit(as.vector(rt[, 2]), " "))

geneNames <- gsub("^ | $", "", geneNames)

uniqGene <- unique(geneNames)

header <- gsub(".csv", "", inputFile)

Ingredient <- admeRT[header, "Ingredient name"]

if (length(uniqGene) > 0) {

outTab <- rbind(outTab, cbind(id = header, Ingredient = Ingredient, Gene = uniqGene))

}

}

write.table(outTab, file = "Drug.txt", sep = "\t", quote = FALSE, row.names = FALSE)

# ==============================================================================

# SECTION 6: Disease Target Collection (GeneCards, OMIM, DisGeNET, TTD)

# ==============================================================================

library(ggvenn)

files <- list.files(pattern = "*.txt$")

geneList <- list()

for (inputFile in files) {

if (inputFile == "Disease.txt") next

rt <- read.table(inputFile, header = FALSE, sep = "\t", check.names = FALSE)

geneNames <- unlist(strsplit(as.vector(rt[, 1]), " "))

geneNames <- gsub("^ | $", "", geneNames)

uniqGene <- unique(geneNames)

header <- unlist(strsplit(inputFile, "\\.|\\-"))[1]

geneList[[header]] <- uniqGene

}

pdf(file = "venn.pdf", width = 6, height = 6)

ggvenn(geneList, show_percentage = TRUE, stroke_color = "white", stroke_size = 0.5,

fill_color = c("#E41A1C", "#1E90FF", "#FF8C00", "#31A354FF"),

set_name_color = c("#E41A1C", "#1E90FF", "#FF8C00", "#31A354FF"),

set_name_size = 6, text_size = 4.5)

dev.off()

unionGenes <- Reduce(union, geneList)

write.table(unionGenes, file = "Disease.txt", sep = "\t", quote = FALSE, col.names = FALSE, row.names = FALSE)

# ==============================================================================

# SECTION 7: Drug-Disease Target Intersection

# ==============================================================================

library(ggvenn)

drugName <- "Fragrant Solomonseal Rhizome"

diseaseName <- "Sjogren's Syndrome"

geneList <- list()

drugRT <- read.table("Drug.txt", header = TRUE, sep = "\t", check.names = FALSE, comment.char = "", quote = "")

geneList[[drugName]] <- as.vector(drugRT[, 3])

disRT <- read.table("Disease.txt", header = FALSE, sep = "\t", check.names = FALSE, comment.char = "", quote = "")

geneList[[diseaseName]] <- as.vector(disRT[, 1])

pdf(file = "venn.pdf", width = 6, height = 6)

ggvenn(geneList, show_percentage = TRUE, stroke_color = "white", stroke_size = 0.5,

fill_color = c("#E41A1C", "#1E90FF"), set_name_color = c("#E41A1C", "#1E90FF"),

set_name_size = 6, text_size = 4.5)

dev.off()

interGenes <- Reduce(intersect, geneList)

write.table(interGenes, file = "interGenes.txt", sep = "\t", quote = FALSE, col.names = FALSE, row.names = FALSE)

interInfo <- drugRT[drugRT$Gene %in% interGenes, ]

interInfo <- cbind(interInfo, drugName = drugName, diseaseName = diseaseName)

write.table(interInfo, file = "interGenes.info.txt", sep = "\t", quote = FALSE, row.names = FALSE)

# ==============================================================================

# SECTION 8: GO Enrichment Analysis

# ==============================================================================

library(clusterProfiler)

library(org.Hs.eg.db)

library(enrichplot)

library(ggplot2)

library(circlize)

library(RColorBrewer)

library(dplyr)

library(ggpubr)

library(ComplexHeatmap)

library(stringr)

pvalueFilter <- 0.05

p.adjustFilter <- 0.05

rt <- read.table("interGenes.txt", header = FALSE, sep = "\t", check.names = FALSE)

genes <- unique(as.vector(rt[, 1]))

entrezIDs <- mget(genes, org.Hs.egSYMBOL2EG, ifnotfound = NA)

entrezIDs <- as.character(entrezIDs)

gene <- entrezIDs[entrezIDs != "NA"]

kk <- enrichGO(gene = gene, OrgDb = org.Hs.eg.db, pvalueCutoff = 1, qvalueCutoff = 1,

ont = "all", readable = TRUE)

GO <- as.data.frame(kk)

GO <- GO[(GO$pvalue < pvalueFilter & GO$p.adjust < p.adjustFilter), ]

write.table(GO, file = "GO.txt", sep = "\t", quote = FALSE, row.names = FALSE)

# Barplot

go_data <- as.data.frame(kk)

go_data <- go_data[go_data$p.adjust < 0.05, ]

go_data <- go_data %>% group_by(ONTOLOGY) %>% slice_head(n = 10)

pdf("barplot_fixed.pdf", width = 13, height = 8)

p <- ggplot(go_data, aes(x = reorder(Description, Count), y = Count, fill = ONTOLOGY)) +

geom_bar(stat = "identity") + coord_flip() +

facet_grid(ONTOLOGY ~ ., scales = "free", space = "free") +

scale_fill_brewer(palette = "Set2") + theme_minimal(base_size = 11) +

theme(strip.text = element_text(size = 12),

axis.text.y = element_text(size = 7, hjust = 1, lineheight = 0.8),

axis.text.x = element_text(size = 10),

plot.title = element_text(size = 14, hjust = 0.5)) +

labs(x = NULL, y = "Gene Count", fill = "Ontology", title = "GO Enrichment (Top 10 per Ontology)") +

scale_x_discrete(labels = function(x) str_wrap(x, width = 45))

print(p)

dev.off()

# Bubble plot

pdf(file = "bubble_v1.pdf", width = 10, height = 7)

bub <- dotplot(kk, showCategory = 10, orderBy = "GeneRatio", label_format = 100,

split = "ONTOLOGY", color = "p.adjust") +

facet_grid(ONTOLOGY ~ ., scale = 'free') +

theme(axis.text.x = element_text(angle = 60, hjust = 1, vjust = 1, size = 6),

axis.text.y = element_text(size = 8),

plot.margin = unit(c(0.5, 0.5, 1, 0.5), "cm"))

print(bub)

dev.off()

# ==============================================================================

# SECTION 9: KEGG Enrichment Analysis

# ==============================================================================

library(clusterProfiler)

library(org.Hs.eg.db)

library(enrichplot)

library(ggplot2)

library(dplyr)

pvalueFilter <- 0.05

p.adjustFilter <- 0.05

rt <- read.table("interGenes.txt", header = FALSE, sep = "\t", check.names = FALSE)

genes <- unique(as.vector(rt[, 1]))

entrezIDs <- mget(genes, org.Hs.egSYMBOL2EG, ifnotfound = NA)

entrezIDs <- as.character(entrezIDs)

rt <- data.frame(genes, entrezID = entrezIDs)

gene <- entrezIDs[entrezIDs != "NA"]

kk <- enrichKEGG(gene = gene, organism = "hsa", pvalueCutoff = 1, qvalueCutoff = 1)

KEGG <- as.data.frame(kk)

KEGG$geneID <- as.character(sapply(KEGG$geneID, function(x)

paste(rt$genes[match(strsplit(x, "/")[[1]], as.character(rt$entrezID))], collapse = "/")))

KEGG <- KEGG[(KEGG$pvalue < pvalueFilter & KEGG$p.adjust < p.adjustFilter), ]

KEGG <- KEGG[KEGG$category != "Human Diseases", ]

KEGG <- na.omit(KEGG)

write.table(KEGG, file = "KEGG.txt", sep = "\t", quote = FALSE, row.names = FALSE)

# Barplot

showNum <- ifelse(nrow(KEGG) > 30, 30, nrow(KEGG))

showKEGG <- KEGG[1:showNum, ]

showKEGG$pathway <- factor(showKEGG$Description, levels = rev(showKEGG$Description))

bar <- ggplot(data = showKEGG, aes(x = Count, y = pathway, fill = pvalue)) +

geom_bar(stat = "identity", width = 0.75) +

scale_fill_distiller(palette = "Spectral", direction = 1) +

labs(x = "Gene count", y = "", title = "Enriched KEGG Pathways") +

theme_bw() + theme(plot.title = element_text(size = 12, hjust = 0.5, face = "bold"))

pdf(file = "barplot.pdf", width = 7, height = 5.5)

print(bar)

dev.off()

# Bubble plot

showKEGG$GeneRatio <- sapply(showKEGG$GeneRatio, function(x) eval(parse(text = x)))

showKEGG <- showKEGG[order(showKEGG$GeneRatio), ]

showKEGG$pathway <- factor(showKEGG$pathway, levels = showKEGG$pathway)

bubble <- ggplot(data = showKEGG, aes(x = GeneRatio, y = pathway)) +

geom_point(aes(size = Count, color = pvalue)) +

scale_color_distiller(palette = "Spectral", direction = 1) +

labs(x = "Gene ratio", y = "", title = "Enriched KEGG Pathways", size = "Count") +

theme_bw() + theme(plot.title = element_text(size = 12, hjust = 0.5, face = "bold"))

pdf(file = "bubble.pdf", width = 7, height = 5.5)

print(bubble)

dev.off()

# ==============================================================================

# SECTION 10: Three-Dimensional Intersection (Drug-Disease-DEG)

# ==============================================================================

library(ggvenn)

drugName <- "Fragrant Solomonseal Rhizome"

diseaseName <- "Sjogren's Syndrome"

geneList <- list()

drugRT <- read.table("Drug.txt", header = TRUE, sep = "\t", check.names = FALSE, comment.char = "", quote = "")

geneList[[drugName]] <- as.vector(drugRT[, 3])

disRT <- read.table("Disease.txt", header = FALSE, sep = "\t", check.names = FALSE, comment.char = "", quote = "")

geneList[[diseaseName]] <- as.vector(disRT[, 1])

diffRT <- read.table("diff.txt", header = TRUE, sep = "\t", check.names = FALSE, comment.char = "", quote = "")

geneList[["DEG"]] <- as.vector(diffRT[, 1])

pdf(file = "venn.pdf", width = 6, height = 6)

ggvenn(geneList, show_percentage = TRUE, stroke_color = "white", stroke_size = 0.5,

fill_color = c("#E41A1C", "#1E90FF", "#FF8C00"),

set_name_color = c("#E41A1C", "#1E90FF", "#FF8C00"),

set_name_size = 6, text_size = 4.5)

dev.off()

interGenes <- Reduce(intersect, geneList)

write.table(interGenes, file = "interGenes.txt", sep = "\t", quote = FALSE, col.names = FALSE, row.names = FALSE)

# ==============================================================================

# SECTION 11: Diagnostic Model (Nomogram, Calibration, DCA, CIC)

# ==============================================================================

library(rms)

library(rmda)

target_genes <- c("CCR1")

gene_matrix <- read.table("merge.normalize.txt", header = TRUE, sep = "\t", row.names = 1, check.names = FALSE)

expr_data <- as.data.frame(t(gene_matrix))

expr_data$group <- ifelse(grepl("_Control", rownames(expr_data)), 0, 1)

expr_data <- expr_data[, c(target_genes, "group")]

expr_data$group <- as.factor(expr_data$group)

pdf("Diagnostic_Model_CCR1.pdf", width = 12, height = 11)

par(mfrow = c(2, 2), mar = c(5, 4, 4, 2) + 0.1)

dd <- datadist(expr_data)

options(datadist = "dd")

lrm_model <- lrm(group ~ CCR1, data = expr_data, x = TRUE, y = TRUE)

# Nomogram

nomo <- nomogram(lrm_model, fun = plogis, funlabel = "Risk of Disease",

fun.at = seq(0.1, 0.9, by = 0.1))

plot(nomo, main = "A: Nomogram (CCR1)", cex.axis = 0.8, cex.var = 0.9, lmgp = 0.25)

# Calibration Curve

cal <- calibrate(lrm_model, method = "boot", B = 1000)

plot(cal, xlab = "Predicted Probability", ylab = "Actual Probability", main = "B: Calibration Curve")

abline(0, 1, col = "red", lty = 2)

# DCA

expr_data$group <- as.numeric(as.character(expr_data$group))

dca_ccr1 <- decision_curve(group ~ CCR1, data = expr_data, family = binomial(link = 'logit'))

plot_decision_curve(list(dca_ccr1), curve.names = c("CCR1 only"),

xlab = "Threshold Probability", main = "C: Decision Curve Analysis (DCA)",

legend.position = "topright")

# CIC

plot_clinical_impact(dca_ccr1, main = "D: Clinical Impact Curve (CIC)", legend.position = "topright")

dev.off()

par(mfrow = c(1, 1))

# ==============================================================================

# SECTION 12: ROC Analysis (External Validation)

# ==============================================================================

library(pROC)

expFile <- "geneMatrix.txt"

sample_group1_file <- "sample1.txt"

sample_group2_file <- "sample2.txt"

genes_to_analyze <- c("CCR1")

expression_matrix_long <- read_tsv(expFile, col_names = TRUE, show_col_types = FALSE)

if (any(duplicated(expression_matrix_long[[1]]))) {

expression_matrix_processed <- expression_matrix_long %>%

group_by(!!sym(names(expression_matrix_long)[1])) %>%

summarise(across(everything(), mean, na.rm = TRUE)) %>%

column_to_rownames(var = names(expression_matrix_long)[1])

} else {

expression_matrix_processed <- expression_matrix_long %>%

column_to_rownames(var = names(expression_matrix_long)[1])

}

data_transposed <- as.data.frame(t(as.matrix(sapply(expression_matrix_processed, as.numeric))))

rownames(data_transposed) <- rownames(expression_matrix_processed)

group1_ids <- readLines(sample_group1_file); group1_ids <- group1_ids[group1_ids != ""]

group2_ids <- readLines(sample_group2_file); group2_ids <- group2_ids[group2_ids != ""]

group_info <- data.frame(sample_id = c(group1_ids, group2_ids),

group_label = c(rep(0, length(group1_ids)), rep(1, length(group2_ids))))

merged_data <- data_transposed %>% rownames_to_column("sample_id") %>% inner_join(group_info, by = "sample_id")

outTab <- data.frame()

for (gene in genes_to_analyze) {

if (!gene %in% colnames(merged_data)) next

roc_obj <- roc(merged_data$group_label, merged_data[[gene]], quiet = TRUE)

set.seed(123)

ci1 <- ci.auc(roc_obj, method = "bootstrap")

ciVec <- as.numeric(ci1)

pdf(file = paste0("ROC_", gene, ".pdf"), width = 5, height = 4.7)

plot(roc_obj, print.auc = TRUE, legacy.axes = TRUE, col = "red", main = gene,

thresholds = "best", print.thres = "best")

text(0.39, 0.43, paste0("95% CI: ", sprintf("%.03f", ciVec[1]), "-", sprintf("%.03f", ciVec[3])), col = "red")

dev.off()

roc_result <- coords(roc_obj, "best", ret = "all", transpose = FALSE)

if (is.data.frame(roc_result)) roc_result <- roc_result[1, ]

outTab <- rbind(outTab, data.frame(Gene = gene,

AUC = paste0(sprintf("%.03f", roc_obj$auc), " (", sprintf("%.03f", ciVec[1]), "-", sprintf("%.03f", ciVec[3]), ")"),

Cutoff = roc_result["threshold"], Sensitivity = roc_result["sensitivity"],

Specificity = roc_result["specificity"]))

}

write.table(outTab, file = "ROC_result_summary.xls", sep = "\t", quote = FALSE, row.names = FALSE)

# ==============================================================================

# SECTION 13: GSEA (Gene Set Enrichment Analysis)

# ==============================================================================

library(limma)

library(org.Hs.eg.db)

library(clusterProfiler)

library(enrichplot)

expFile <- "merge.normalize.txt"

geneFile <- "interFeatureGenes.txt"

gmtFile <- "c2.cp.kegg.Hs.symbols.gmt"

rt <- read.table(expFile, header = TRUE, sep = "\t", check.names = FALSE)

rt <- as.matrix(rt); rownames(rt) <- rt[, 1]

exp <- rt[, 2:ncol(rt)]

dimnames <- list(rownames(exp), colnames(exp))

data <- matrix(as.numeric(as.matrix(exp)), nrow = nrow(exp), dimnames = dimnames)

data <- avereps(data)

data <- data[rowMeans(data) > 0, ]

Type <- gsub("(.*)\\_(.*)\\_(.*)", "\\3", colnames(data))

data <- data[, Type == "Treat", drop = FALSE]

geneRT <- read.table(geneFile, header = FALSE, sep = "\t", check.names = FALSE)

for (gene in as.vector(geneRT[, 1])) {

dataL <- data[, data[gene, ] < median(data[gene, ]), drop = FALSE]

dataH <- data[, data[gene, ] >= median(data[gene, ]), drop = FALSE]

meanL <- rowMeans(dataL); meanH <- rowMeans(dataH)

meanL[meanL < 0.00001] <- 0.00001; meanH[meanH < 0.00001] <- 0.00001

logFC <- sort(meanH - meanL, decreasing = TRUE)

gmt <- read.gmt(gmtFile)

kk <- GSEA(logFC, TERM2GENE = gmt, pvalueCutoff = 1)

kkTab <- as.data.frame(kk)

kkTab <- kkTab[kkTab$pvalue < 0.05, ]

write.table(kkTab, file = paste0(gene, ".result.txt"), sep = "\t", quote = FALSE, row.names = FALSE)

# High expression group

termNum <- 5

kkUp <- kkTab[kkTab$NES > 0, ]

termNum <- ifelse(nrow(kkUp) >= termNum, termNum, nrow(kkUp))

if (termNum > 0) {

showTerm <- row.names(kkUp)[1:termNum]

gseaplot <- gseaplot2(kk, showTerm, base_size = 8, title = paste0("Enriched in high ", gene, " group"))

pdf(file = paste0(gene, ".highExp.pdf"), width = 6.5, height = 5.5); print(gseaplot); dev.off()

}

# Low expression group

termNum <- 5

kkDown <- kkTab[kkTab$NES < 0, ]

termNum <- ifelse(nrow(kkDown) >= termNum, termNum, nrow(kkDown))

if (termNum > 0) {

showTerm <- row.names(kkDown)[1:termNum]

gseaplot <- gseaplot2(kk, showTerm, base_size = 8, title = paste0("Enriched in low ", gene, " group"))

pdf(file = paste0(gene, ".lowExp.pdf"), width = 6.5, height = 5.5); print(gseaplot); dev.off()

}

}

# ==============================================================================

# SECTION 14: CIBERSORT Immune Cell Infiltration Analysis

# ==============================================================================

inputFile <- "merge.normalize.txt"

source("CIBERSORT.R") # Load CIBERSORT function

outTab <- CIBERSORT("ref.txt", inputFile, perm = 1000)

outTab <- outTab[outTab[, "P-value"] < 0.05, ]

outTab <- as.matrix(outTab[, 1:(ncol(outTab) - 3)])

outTab <- rbind(id = colnames(outTab), outTab)

write.table(outTab, file = "CIBERSORT-Results.txt", sep = "\t", quote = FALSE, col.names = FALSE)

# ==============================================================================

# SECTION 15: Immune Cell Infiltration Visualization

# (Barplot, Boxplot, Correlation Heatmap)

# ==============================================================================

library(reshape2)

library(ggpubr)

library(corrplot)

inputFile <- "CIBERSORT-Results.txt"

rt <- read.table(inputFile, header = TRUE, sep = "\t", check.names = FALSE, row.names = 1)

con <- grepl("_Control", rownames(rt), ignore.case = TRUE)

treat <- grepl("_Treat", rownames(rt), ignore.case = TRUE)

conData <- rt[con, ]; treatData <- rt[treat, ]

conNum <- nrow(conData); treatNum <- nrow(treatData)

data <- t(rbind(conData, treatData))

# Stacked barplot

pdf(file = "barplot.pdf", width = 13, height = 7)

col <- rainbow(nrow(data), s = 0.7, v = 0.7)

par(las = 1, mar = c(8, 5, 4, 16), mgp = c(3, 0.1, 0), cex.axis = 1.5)

a1 <- barplot(data, col = col, xaxt = "n", yaxt = "n", ylab = "Relative Percent", cex.lab = 1.5)

a2 <- axis(2, tick = FALSE, labels = FALSE)

axis(2, a2, paste0(a2 * 100, "%"))

par(srt = 0, xpd = TRUE)

rect(xleft = a1[1] - 0.5, ybottom = -0.01, xright = a1[conNum] + 0.5, ytop = -0.08, col = "#6699FFFF")

text(a1[conNum] / 2, -0.045, "Control", cex = 1.8)

rect(xleft = a1[conNum] + 0.5, ybottom = -0.01, xright = a1[length(a1)] + 0.5, ytop = -0.08, col = "#E6550DFF")

text((a1[length(a1)] + a1[conNum]) / 2, -0.045, "Treat", cex = 1.8)

legend(par('usr')[2] * 0.98, par('usr')[4], legend = rownames(data), col = col, pch = 15, bty = "n", cex = 1)

dev.off()

# Boxplot

Type <- gsub("(.*)\\_(.*)", "\\2", colnames(data))

data_box <- cbind(as.data.frame(t(data)), Type)

data_box <- melt(data_box, id.vars = c("Type"))

colnames(data_box) <- c("Type", "Immune", "Expression")

bioCol <- c("#6699FFFF", "#E6550DFF")

boxplot <- ggboxplot(data_box, x = "Immune", y = "Expression", fill = "Type",

xlab = "", ylab = "Fraction", legend.title = "Type", width = 0.8, palette = bioCol) +

rotate_x_text(50) +

stat_compare_means(aes(group = Type), symnum.args = list(cutpoints = c(0, 0.001, 0.01, 0.05, 1),

symbols = c("***", "**", "*", "")), label = "p.signif")

pdf(file = "immune.diff.pdf", width = 8, height = 6); print(boxplot); dev.off()

# Correlation heatmap

treatData <- treatData[, apply(treatData, 2, sd) > 0]

pdf(file = "corHeatmap.pdf", width = 12, height = 12)

corrplot(corr = cor(treatData, method = "spearman"), method = "color", order = "hclust",

tl.col = "black", number.cex = 0.8, addCoef.col = "black",

col = colorRampPalette(c("#6699FFFF", "white", "#E6550DFF"))(50))

dev.off()

# ==============================================================================

# SECTION 16: CCR1-Immune Cell Correlation (Lollipop + Heatmap + Network)

# ==============================================================================

library(limma)

library(reshape2)

library(ggpubr)

library(ggExtra)

expFile <- "merge.normalize.txt"

geneFile <- "interFeatureGenes.txt"

immFile <- "CIBERSORT-Results.txt"

rt <- read.table(expFile, header = TRUE, sep = "\t", check.names = FALSE)

rt <- as.matrix(rt); rownames(rt) <- rt[, 1]

exp <- rt[, 2:ncol(rt)]

dimnames <- list(rownames(exp), colnames(exp))

data <- matrix(as.numeric(as.matrix(exp)), nrow = nrow(exp), dimnames = dimnames)

data <- avereps(data)

Type <- gsub("(.*)\\_(.*)\\_(.*)", "\\3", colnames(data))

allData <- data[, Type == "Treat", drop = FALSE]

geneRT <- read.table(geneFile, header = FALSE, sep = "\t", check.names = FALSE)

for (gene in as.vector(geneRT[, 1])) {

data_gene <- t(allData[gene, , drop = FALSE])

data_gene <- as.data.frame(data_gene)

immune <- read.table(immFile, header = TRUE, sep = "\t", check.names = FALSE, row.names = 1)

sameSample <- intersect(row.names(immune), row.names(data_gene))

rt_merged <- cbind(immune[sameSample, , drop = FALSE], data_gene[sameSample, , drop = FALSE])

outTab <- data.frame()

for (i in colnames(rt_merged)[1:(ncol(rt_merged) - 1)]) {

x <- as.numeric(rt_merged[, gene])

y <- as.numeric(rt_merged[, i])

if (sd(y) == 0) y[1] <- 0.00001

cor_result <- cor.test(x, y, method = "spearman")

outTab <- rbind(outTab, cbind(Gene = gene, Cell = i, cor = cor_result$estimate, pvalue = cor_result$p.value))

}

write.table(outTab, file = paste0("corr.", gene, ".result.txt"), sep = "\t", row.names = FALSE, quote = FALSE)

}

# ==============================================================================

# SECTION 17: GSVA Pathway Activity Analysis

# ==============================================================================

library(reshape2)

library(ggpubr)

library(limma)

library(GSEABase)

library(GSVA)

gene <- "CCR1"

expFile <- "merge.normalize.txt"

gmtFile <- "c2.cp.kegg.Hs.symbols.gmt"

rt <- read.table(expFile, header = TRUE, sep = "\t", check.names = FALSE)

rt <- as.matrix(rt); rownames(rt) <- rt[, 1]

exp <- rt[, 2:ncol(rt)]

dimnames <- list(rownames(exp), colnames(exp))

data <- matrix(as.numeric(as.matrix(exp)), nrow = nrow(exp), dimnames = dimnames)

data <- avereps(data)

Type <- gsub("(.*)\\_(.*)\\_(.*)", "\\3", colnames(data))

data <- data[, Type == "Treat", drop = FALSE]

geneSets <- getGmt(gmtFile, geneIdType = SymbolIdentifier())

# GSVA analysis (try multiple methods for version compatibility)

tryCatch({

gsva_param <- ssgseaParam(exprData = data, geneSets = geneSets)

gsvaScore <- gsva(gsva_param)

}, error = function(e) {

tryCatch({

gsvaScore <<- gsva(data, geneSets, method = 'ssgsea', kcdf = 'Gaussian', abs.ranking = TRUE)

}, error = function(e2) {

gsvaScore <<- gsva(data, geneSets)

})

})

normalize <- function(x) (x - min(x)) / (max(x) - min(x))

gsvaScore <- normalize(gsvaScore)

gsvaScore <- gsvaScore[apply(gsvaScore, 1, sd) > 0.01, ]

lowName <- colnames(data)[data[gene, ] < median(data[gene, ])]

highName <- colnames(data)[data[gene, ] >= median(data[gene, ])]

lowScore <- gsvaScore[, lowName]

highScore <- gsvaScore[, highName]

data_T_test <- cbind(lowScore, highScore)

Type_T_test <- c(rep("Control", ncol(lowScore)), rep("Treat", ncol(highScore)))

outTab <- data.frame()

for (i in row.names(data_T_test)) {

test <- t.test(data_T_test[i, ] ~ Type_T_test)

t_val <- test$statistic

pvalue <- test$p.value

if (test$estimate[2] > test$estimate[1]) t_val <- abs(t_val) else t_val <- -abs(t_val)

Sig <- ifelse(pvalue > 0.05, "Not", ifelse(t_val > 0, "Up", "Down"))

outTab <- rbind(outTab, cbind(Pathway = i, t = t_val, pvalue = pvalue, Sig = Sig))

}

# Select pathways for plotting

notSigTab <- outTab[outTab$Sig == "Not", ]; notSigTab <- notSigTab[order(as.numeric(notSigTab$t)), ]

sigTab <- outTab[outTab$Sig != "Not", ]; sigTab <- sigTab[order(as.numeric(sigTab$t)), ]

if (nrow(sigTab) > 20) {

outTab <- rbind(sigTab[c(1:10, ((nrow(sigTab) - 9):nrow(sigTab))), ],

notSigTab[c(1:5, ((nrow(notSigTab) - 4):nrow(notSigTab))), ])

} else {

outTab <- rbind(sigTab, notSigTab[c(1:5, ((nrow(notSigTab) - 4):nrow(notSigTab))), ])

}

pdf(file = "barplot.pdf", width = 10.5, height = 7)

outTab$t <- as.numeric(outTab$t)

outTab$Sig <- factor(outTab$Sig, levels = c("Down", "Not", "Up"))

gg1 <- ggbarplot(outTab, x = "Pathway", y = "t", fill = "Sig", color = "white",

palette = c("green3", "grey", "red3"), sort.val = "asc", sort.by.groups = TRUE,

rotate = TRUE, title = gene, legend.title = "Group", legend = "right",

xlab = "", ylab = "t value of GSVA score", x.text.angle = 60)

print(gg1)

dev.off()

# ==============================================================================

# SECTION 18: Single-Cell RNA Sequencing Analysis (Seurat + Harmony)

# Input: GSE253568 (9 pSS + 8 HC PBMCs)

# ==============================================================================

library(limma)

library(Seurat)

library(dplyr)

library(magrittr)

library(celldex)

library(SingleR)

library(monocle)

library(clustree)

library(harmony)

logFCfilter <- 1

adjPvalFilter <- 0.05

# Read 10X data

dirs <- list.dirs()

dirs_sample <- dirs[-1]

names(dirs_sample) <- gsub(".+\\/(.+)", "\\1", dirs_sample)

counts <- Read10X(data.dir = dirs_sample)

pbmc <- CreateSeuratObject(counts, min.cells = 5, min.features = 200)

# QC: mitochondrial gene percentage

pbmc[["percent.mt"]] <- PercentageFeatureSet(object = pbmc, pattern = "^MT-")

pdf(file = "01.featureViolin.pdf", width = 10, height = 6.5)

VlnPlot(object = pbmc, features = c("nFeature_RNA", "nCount_RNA", "percent.mt"), ncol = 3)

dev.off()

pbmc <- subset(x = pbmc, subset = nFeature_RNA > 100 & percent.mt < 15)

# Normalization

pbmc <- NormalizeData(object = pbmc, normalization.method = "LogNormalize", scale.factor = 10000)

pbmc <- FindVariableFeatures(object = pbmc, selection.method = "vst", nfeatures = 1500)

# PCA + Harmony batch correction

pbmc <- ScaleData(pbmc)

pbmc <- RunPCA(object = pbmc, npcs = 20, pc.genes = VariableFeatures(object = pbmc))

pbmc <- RunHarmony(pbmc, "orig.ident")

# Clustering

pcSelect <- 20

pbmc <- FindNeighbors(object = pbmc, dims = 1:pcSelect)

pbmc <- FindClusters(object = pbmc, resolution = 0.6)

# t-SNE visualization

pbmc <- RunTSNE(object = pbmc, dims = 1:pcSelect)

pdf(file = "03.cluster.pdf", width = 7.5, height = 6)

TSNEPlot(object = pbmc, pt.size = 2, label = TRUE)

dev.off()

# Find cluster markers

pbmc.markers <- FindAllMarkers(object = pbmc, only.pos = FALSE, min.pct = 0.25, logfc.threshold = logFCfilter)

sig.markers <- pbmc.markers[(abs(as.numeric(as.vector(pbmc.markers$avg_log2FC))) > logFCfilter &

as.numeric(as.vector(pbmc.markers$p_val_adj)) < adjPvalFilter), ]

write.table(sig.markers, file = "03.clusterMarkers.txt", sep = "\t", row.names = FALSE, quote = FALSE)

# Cell type annotation with SingleR (multiple references)

pbmc_for_SingleR <- GetAssayData(pbmc, layer = "data")

clusters <- pbmc@meta.data$seurat_clusters

# Load reference datasets (ref_Human_all.RData, ref_Hematopoietic.RData, etc.)

singler <- SingleR(test = pbmc_for_SingleR,

ref = list(ref1, ref2, ref3, ref4, ref5, ref6, ref7),

labels = list(ref1$label.main, ref2$label.main, ref3$label.main,

ref4$label.main, ref5$label.main, ref6$label.main, ref7$label.main),

clusters = clusters)

singler$labels <- gsub("_|-", " ", singler$labels)

clusterAnn <- as.data.frame(singler)

clusterAnn <- cbind(id = row.names(clusterAnn), clusterAnn)

clusterAnn <- clusterAnn[, c("id", "labels")]

write.table(clusterAnn, file = "04.clusterAnn.txt", quote = FALSE, sep = "\t", row.names = FALSE)

# Rename identities and visualize

newLabels <- singler$labels

names(newLabels) <- levels(pbmc)

pbmc <- RenameIdents(pbmc, newLabels)

pdf(file = "04.cellAnn.pdf", width = 7.5, height = 6)

TSNEPlot(object = pbmc, pt.size = 2, label = TRUE)

dev.off()

# Group visualization

Type <- gsub("(.*?)\\..*", "\\1", colnames(pbmc))

names(Type) <- colnames(pbmc)

pbmc <- AddMetaData(object = pbmc, metadata = Type, col.name = "Type")

# Save Seurat object

save(pbmc, cellAnn, file = "Seurat.Rdata")

# ==============================================================================

# SECTION 19: CellChat Cell Communication Analysis

# ==============================================================================

library(CellChat)

library(Seurat)

library(NMF)

library(ggplot2)

library(ggalluvial)

load("Seurat.Rdata")

expMatrix <- as.matrix(GetAssayData(pbmc, assay = "RNA", layer = "data"))

meta <- read.table("04.cellAnn.txt", header = TRUE, sep = "\t", check.names = FALSE, row.names = 1)

colnames(meta)[1] <- "labels"

common_cells <- intersect(colnames(expMatrix), rownames(meta))

expMatrix <- expMatrix[, common_cells]

meta <- meta[common_cells, , drop = FALSE]

cellchat <- createCellChat(object = expMatrix, meta = meta, group.by = "labels")

cellchat <- setIdent(cellchat, ident.use = "labels")

groupSize <- as.numeric(table(cellchat@idents))

CellChatDB.use <- subsetDB(CellChatDB.human, search = "Secreted Signaling")

cellchat@DB <- CellChatDB.use

cellchat <- subsetData(cellchat)

cellchat <- identifyOverExpressedGenes(cellchat)

cellchat <- identifyOverExpressedInteractions(cellchat)

cellchat <- computeCommunProb(cellchat)

cellchat <- filterCommunication(cellchat, min.cells = 10)

cellchat <- computeCommunProbPathway(cellchat)

cellchat <- aggregateNet(cellchat)

# Network visualization

pdf("COMM03_NetworkCount.pdf", width = 7, height = 7)

netVisual_circle(cellchat@net$count, vertex.weight = groupSize, weight.scale = TRUE,

label.edge = FALSE, title.name = "Number of interactions")

dev.off()

pdf("COMM04_NetworkWeight.pdf", width = 7, height = 7)

netVisual_circle(cellchat@net$weight, vertex.weight = groupSize, weight.scale = TRUE,

label.edge = FALSE, title.name = "Interaction strength")

dev.off()

# MIF pathway analysis

cellchat <- netAnalysis_computeCentrality(cellchat, slot.name = "netP")

if ("MIF" %in% cellchat@netP$pathways) {

pdf("MIF_Contribution.pdf", width = 8, height = 6)

netAnalysis_contribution(cellchat, signaling = "MIF")

dev.off()

pdf("MIF_Circle.pdf", width = 8, height = 8)

netVisual_aggregate(cellchat, signaling = "MIF", layout = "circle")

dev.off()

}

# ==============================================================================

# SECTION 20: scTenifoldKnk Virtual CCR1 Knockout Analysis

# ==============================================================================

library(Seurat)

library(scTenifoldKnk)

library(ggplot2)

library(ggrepel)

load("Seurat.Rdata")

target_group <- "Pss_Monocytes"

gene_ko <- "CCR1"

subset_cells <- subset(pbmc, subset = group == target_group)

raw_counts <- GetAssayData(subset_cells, slot = "counts")

# Filter low-expression genes

min_cells <- ncol(raw_counts) * 0.05

keep_genes <- rowSums(raw_counts > 0) > min_cells

keep_genes[gene_ko] <- TRUE

countMatrix_filt <- raw_counts[keep_genes, ]

real_gene_names <- rownames(countMatrix_filt)

# Run scTenifoldKnk

result_ko <- scTenifoldKnk(

countMatrix = countMatrix_filt,

gKO = gene_ko,

qc_mtThreshold = 0.25,

qc_minLSize = 500,

nc_nNet = 10,

nc_nCells = 500,

nc_nComp = 3

)

# Fix gene names and export

diff_reg <- result_ko$diffRegulation

if (nrow(diff_reg) == length(real_gene_names)) {

rownames(diff_reg) <- real_gene_names

diff_reg$gene <- real_gene_names

}

diff_reg$log_pval <- -log10(diff_reg$p.adj)

max_val <- max(diff_reg$log_pval[is.finite(diff_reg$log_pval)], na.rm = TRUE)

diff_reg$log_pval[is.infinite(diff_reg$log_pval)] <- max_val + 2

write.csv(diff_reg, paste0("Final_", gene_ko, "_KO_Genes.csv"), row.names = FALSE)

# Barplot: Top 20 affected genes

top_genes <- head(diff_reg[order(-abs(diff_reg$FC)), ], 20)

p1 <- ggplot(top_genes, aes(x = reorder(gene, FC), y = FC)) +

geom_bar(stat = 'identity', fill = "steelblue", width = 0.7) + coord_flip() +

labs(title = paste("Effect of", gene_ko, "Knockout in Pss Monocytes"),

subtitle = "Top 20 Predicted Regulated Genes", x = "Gene Symbol", y = "Fold Change (Predicted)") +

theme_minimal() + theme(plot.title = element_text(face = "bold", size = 14),

axis.text.y = element_text(size = 11, face = "italic", color = "black"))

ggsave(paste0("Final_", gene_ko, "_Barplot.pdf"), p1, width = 8, height = 7)

# Volcano plot

sig_genes <- subset(diff_reg, abs(Z) > 2 & p.adj < 0.05)

top_labels <- head(sig_genes[order(sig_genes$p.adj), ], 15)

p2 <- ggplot(diff_reg, aes(x = Z, y = log_pval)) +

geom_point(color = "grey85", alpha = 0.5, size = 1.5) +

geom_point(data = subset(diff_reg, Z > 2 & p.adj < 0.05), color = "#E41A1C", alpha = 0.8, size = 2) +

geom_point(data = subset(diff_reg, Z < -2 & p.adj < 0.05), color = "#377EB8", alpha = 0.8, size = 2) +

geom_hline(yintercept = -log10(0.05), linetype = "dashed", color = "grey50") +

geom_vline(xintercept = c(-2, 2), linetype = "dashed", color = "grey50") +

geom_text_repel(data = top_labels, aes(label = gene), size = 3.5, fontface = "italic",

box.padding = 0.5, max.overlaps = 20) +

labs(title = paste("Volcano Plot:", gene_ko, "Knockout"),

x = "Z-score (Perturbation Score)", y = "-log10(Adjusted P-value)") +

theme_classic() + theme(plot.title = element_text(face = "bold", size = 14))

ggsave(paste0("Final_", gene_ko, "_Volcano.pdf"), p2, width = 9, height = 7)

# ==============================================================================

# SECTION 21: Molecular Docking (AutoDock Vina)

# Performed using AutoDock Vina v1.2.0 with exhaustiveness = 32

# Receptor: CCR1 protein structure

# Ligand: Moupinamide (from P. odoratum)

# Results visualized using PyMOL and Discovery Studio Visualizer

# ==============================================================================

# Note: Molecular docking was performed using standalone software (AutoDock Vina).

# The docking parameters, grid box settings, and visualization steps are described

# in the Methods section of the manuscript.

################################################################################

# END OF SUPPLEMENTARY CODE

################################################################################
