# Supplementary material for: CCR1-mediated monocyte chemotaxis in the immunopathology of primary Sjögren’s syndrome: multi-omics integration analysis and computational target prioritization implicating Polygonatum odoratum
Source: Front Immunol. 2026 Jul 20;17:1867098. doi: 10.3389/fimmu.2026.1867098 (PMC13429757; doi:10.3389/fimmu.2026.1867098)
Supplement: Supplementary file 5 [file DataSheet5.pdf]

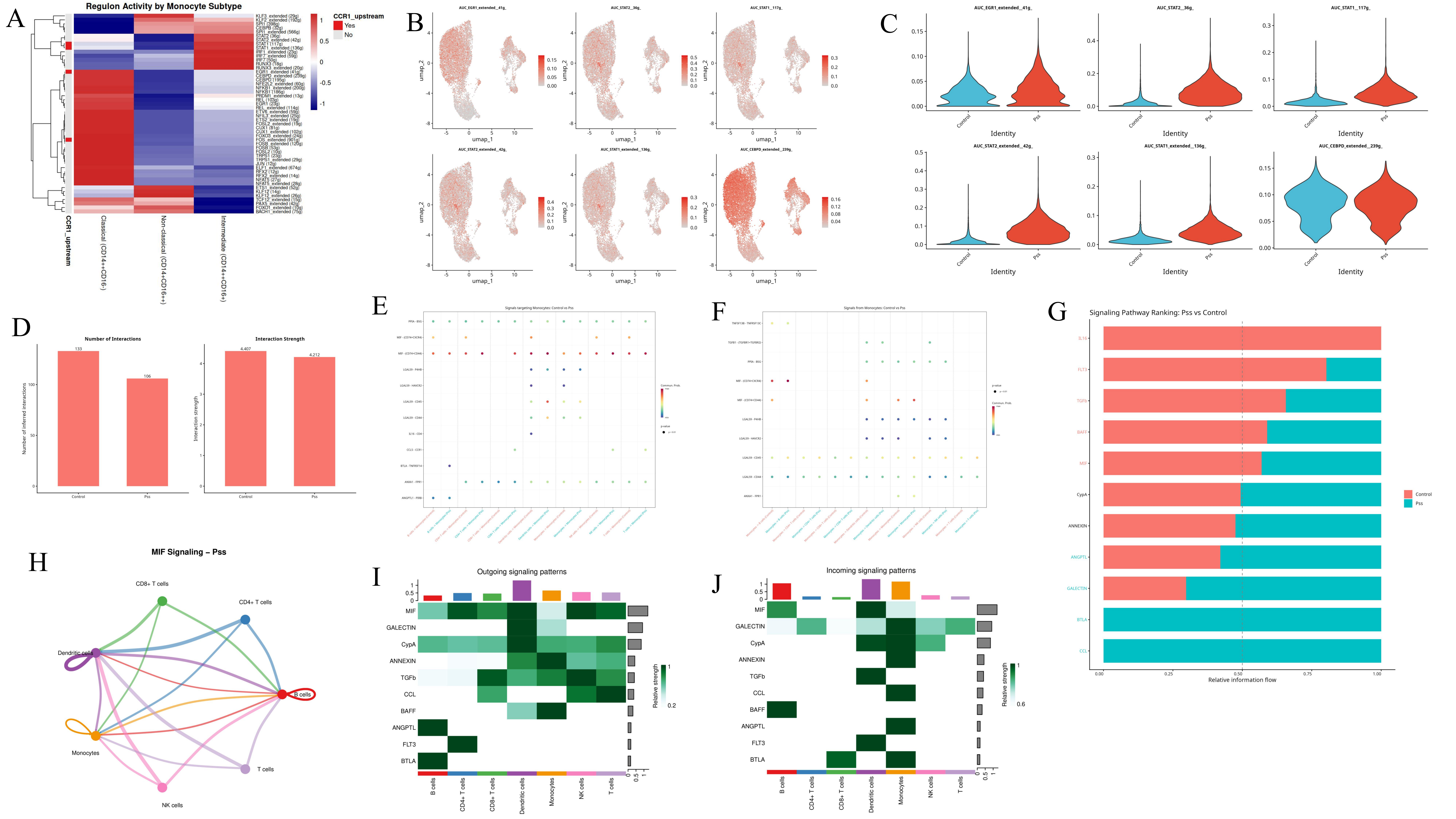

A
Regulon Activity by Monocyte Subtype
CCR1_upstream
Yes
No
CCR1_upstream
Classical (CD14++CD16-)
Non-classical (CD14+CD16++)
Intermediate (CD14++CD16+)
B
C
Identity
Control
Pss
D
Number of Interactions
Interaction Strength
Number of inferred interactions
Interaction strength
133
106
4.407
4.212
E
Signals targeting Monocytes: Control vs Pss
F
Signals from Monocytes: Control vs Pss
G
Signaling Pathway Ranking: Pss vs Control
Relative information flow
IL16
FLT3
TGFb
BAFF
MIF
CypA
ANNEXIN
ANGPTL
GALECTIN
BTLA
CCL
H
MIF Signaling – Pss
CD8+ T cells
CD4+ T cells
Dendritic cells
B cells
Monocytes
T cells
NK cells
I
Outgoing signaling patterns
J
Incoming signaling patterns
Relative strength
